# The Upstream Sequence Transcription Complex dictates nucleosome positioning and promoter accessibility at piRNA genes in the *C*. *elegans* germ line

**DOI:** 10.1371/journal.pgen.1011345

**Published:** 2024-07-10

**Authors:** Nancy Paniagua, C. Jackson Roberts, Lauren E. Gonzalez, David Monedero-Alonso, Valerie Reinke

**Affiliations:** Department of Genetics, Yale University School of Medicine, New Haven Connecticut, United States of America; University of Oxford Department of Biochemistry, UNITED KINGDOM OF GREAT BRITAIN AND NORTHERN IRELAND

## Abstract

The piRNA pathway is a conserved germline-specific small RNA pathway that ensures genomic integrity and continued fertility. In *C*. *elegans* and other nematodes, Type-I piRNAs are expressed from >10,000 independently transcribed genes clustered within two discrete domains of 1.5 and 3.5 MB on Chromosome IV. Clustering of piRNA genes contributes to their germline-specific expression, but the underlying mechanisms are unclear. We analyze isolated germ nuclei to demonstrate that the piRNA genomic domains are located in a heterochromatin-like environment. USTC (Upstream Sequence Transcription Complex) promotes strong association of nucleosomes throughout piRNA clusters, yet organizes the local nucleosome environment to direct the exposure of individual piRNA genes. Localization of USTC to the piRNA domains depends upon the ATPase chromatin remodeler ISW-1, which maintains high nucleosome density across piRNA clusters and ongoing production of piRNA precursors. Overall, this work provides insight into how chromatin states coordinate transcriptional regulation over large genomic domains, with implications for global genome organization.

## Author summary

Here we address how gene expression is coordinated over long stretches of DNA that is embedded in a dense chromatin environment. We focus on a large DNA domain that contains thousands of tiny noncoding piRNA genes that are only expressed in germ cells. Our work demonstrates that a specific factor called USTC works with chromatin remodeling factors and with the core transcription complex to activate piRNA gene expression. The ISW-1 chromatin remodeler creates a dense environment that is favorable for USTC to be recruited to piRNA genes. USTC then clears the chromatin away from a space around each piRNA gene that allows for the factor TBP-1 to bind and presumably recruit the core transcriptional machinery to turn on the short piRNA. This work illuminates a mechanism by which both local and long-distance regulatory information can be integrated to coordinate gene expression.

## Introduction

PIWI-interacting RNAs (piRNAs) are part of a conserved small RNA pathway that maintains germline integrity and fidelity in many animal species. piRNAs silence aberrant expression of foreign genetic elements by associating with the PIWI sub-family of Argonaute proteins [1,2] and targeting transcripts via antisense complementarity, which triggers co-transcriptional or post-transcriptional repression mechanisms [3]. In *C*. *elegans*, there are over 10,000 sequence-diverse piRNAs [4,5]. Mature piRNAs are 21 nucleotides in length, have a strong 5′ uridine bias, and are preferentially expressed in the germline.

Unlike Drosophila or mammals, *C*. *elegans* piRNAs are encoded as individual genes that are transcribed by a paused form of RNA polymerase II into ~28 or ~48 nucleotide precursor RNAs [4–6]. The two types of *C*. *elegans* piRNAs are defined by genome organization: Type-I piRNAs are located within two large clusters on chromosome IV, spanning 1.5 Mb and 3.5 Mb respectively (totaling ~5% of the genome), whereas individual Type-II piRNAs are interspersed throughout the genome, often near promoters of coding genes [4,7,8]. Clustering of Type-I piRNA genes is conserved across nematode species [9,10], implying that clustering facilitates expression.

The transcription of Type-I, but not Type-II, piRNA precursors requires the Upstream Sequence Transcription Complex (USTC) comprising PRDE-1, SNPC-4, TOFU-4, and TOFU-5 [11–13]. The USTC strongly associates with piRNA gene clusters, preferentially binding at the piRNA-specific Ruby motif upstream of piRNA promoters [4,11]. Each component of the complex must be present for the others to concentrate within piRNA clusters [11,12]. While SNPC-4 and TOFU-5 have been implicated in other functions and have binding sites throughout the genome, TOFU-4 and PRDE-1 are expressed specifically in the germ line and bind and function primarily at piRNA clusters [11,12,14]. Intriguingly, both SNPC-4 and TOFU-4 contain SANT domains, suggesting that they have the capacity to bind histones or histone modifications [11,15]. However, the precise mechanisms by which USTC promotes piRNA expression are incompletely understood.

Some clues can be gleaned from proteins beyond the core USTC complex that have also been implicated in piRNA expression, including H3K27me3 methyltransferases and the H3K27me3 reader UAD-2 (USTC Association Dependent 2) [10,16]. Along with ChIP-seq data from whole worms that shows enriched H3K27me3 at piRNA clusters [10], these data suggest that a repressive chromatin environment is important for piRNA transcription. Additionally, the ATPase chromatin remodeler ISW-1, which acts to assemble nucleosomes and establish inter-nucleosomal spacing, has also been implicated in piRNA expression, although how and whether ISW-1 directly affects USTC function is unknown [16]. Notably, USTC physically interacts with TBP-1 (TATA box-binding protein 1) [11]. TBP-1 binds the TATA box found at the promoters of many genes and recruits the RNA polymerase II complex and accessory proteins to initiate transcription. Thus, USTC function is linked with both chromatin- and transcription-associated proteins, although the manner and order in which these factors interact to robustly express piRNAs are still unclear.

Here, we isolate germ nuclei to specifically interrogate the chromatin environment of the genomic domains containing piRNA clusters in *C*. *elegans* germ cells. We show that these domains are indeed enriched for H3K27me3 and contain dense, tightly associated nucleosomes. We demonstrate that continuous ISW-1 activity is required to establish the nucleosome dense neighborhood at piRNA domains, to recruit USTC and to drive Type-I piRNA transcription. USTC directly affects chromatin organization by locally modulating chromatin accessibility around piRNA transcription start sites (TSSs) and by positioning and stabilizing a well-defined nucleosome just upstream of the piRNA TSS, which in turn permits stable association of TBP-1 to enable recruitment of RNA polymerase II. Together, our data show that the germline-specific USTC establishes organized nucleosomes that permit local transcription of individual piRNA genes within an otherwise repressive environment. These structured, accessible micro-domains within a large repressive environment likely facilitate RNA pol II pausing and limit transcription elongation to encourage production of short piRNA precursor transcripts. This mechanism therefore coordinates regulation of thousands of noncoding RNA genes across a multi-megabase domain in a tissue-specific manner.

## Results

### USTC is required to concentrate TBP-1 at piRNA clusters

**Fig 1:**
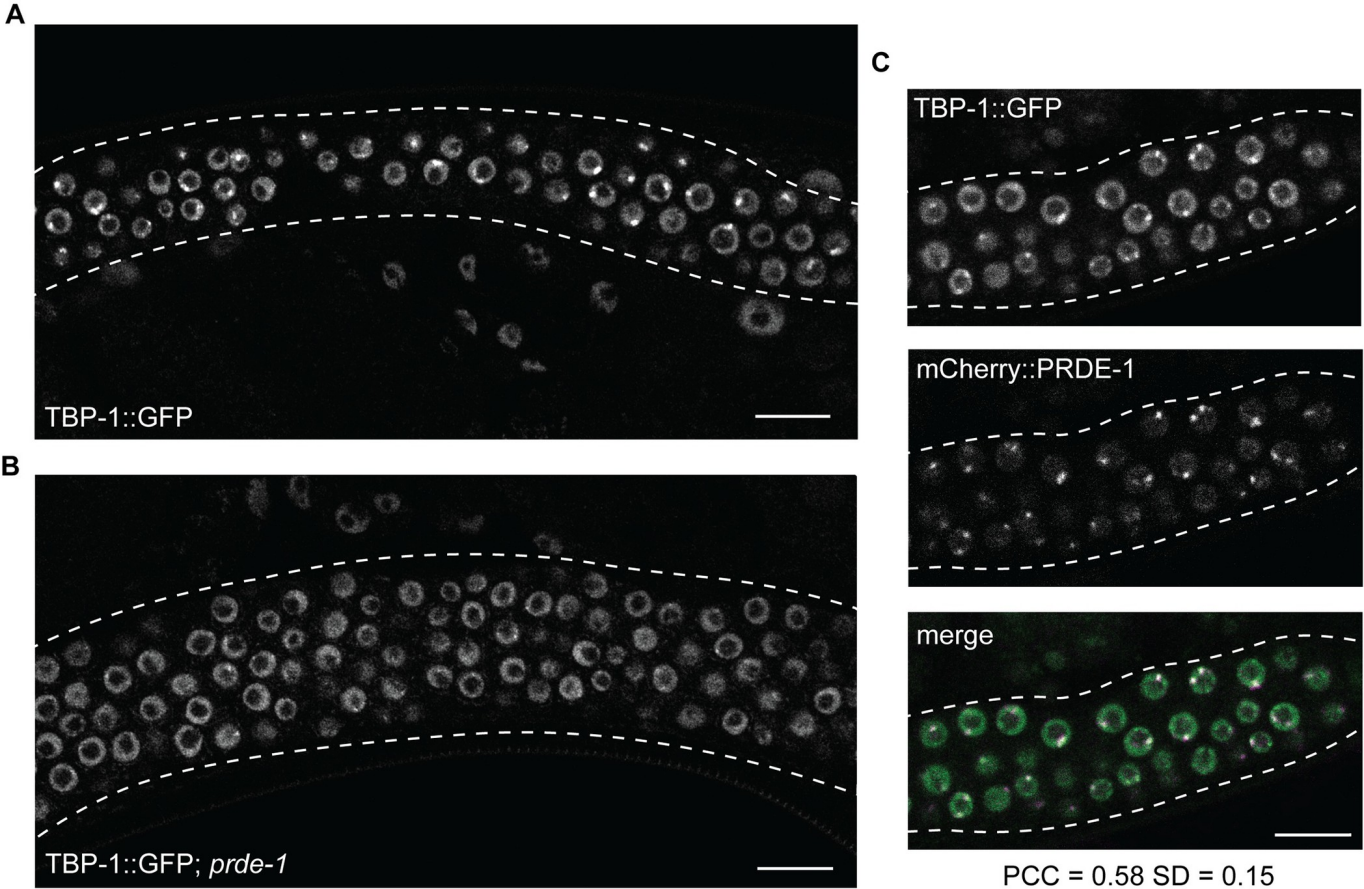
TBP-1 forms PRDE-1-dependent foci at piRNA domains. A) TBP-1:GFP expression in wild type germ cells. B) TBP-1:GFP expression in *prde-1* mutant germ cells. C) Overlap of TBP-1:GFP and mCherry:PRDE-1 foci. Scale bar = 10μm.

Association of USTC with Type-I piRNA clusters in the adult hermaphrodite germ line can be easily detected by fluorescence microscopy as subnuclear foci, and focus formation is often used as a readout of USTC activity [11–13,16,17]. Previously, TBP-1 was demonstrated to physically interact with USTC and is required for USTC foci to form, although it did not form foci itself [11]. To investigate the relationship between TBP-1 and USTC further, we independently examined TBP-1 localization in the germ line using super-resolution microscopy of an existing TBP-1::GFP strain [18]. In contrast to the previous observation, we found that TBP-1 did indeed form foci in germ nuclei, typically one or two strong foci, with additional faint foci per nucleus (Fig 1A). Moreover, the most prominent TBP-1::GFP foci are greatly reduced in animals lacking *prde-1*, a germline-specific component of USTC required for USTC focus formation and production of piRNA precursor transcripts [11–13] (Fig 1B). Because mutating *prde-1* is both required for and specific to USTC formation and function, we refer to USTC rather than *prde-1* when discussing complex activity. We then examined whether TBP-1 foci overlapped with USTC foci using mCherry-tagged PRDE-1. PRDE-1::mCherry foci and and the strongest TBP-1:GFP foci in each nucleus do indeed overlap, while the fainter TBP-1::GFP foci do not (Pearson’s correlation value of 0.58±0.15; Fig 1C). In sum, these data show that USTC and TBP-1 are mutually dependent for focus formation at piRNA domains and presumably for piRNA expression. This finding implies that USTC creates an environment conducive to stable TBP-1 occupancy at piRNA promoters, which acts upstream of RNA pol II recruitment.

### Local, germline-specific depletion of H3K27me3 specifically at piRNA genes within H3K27me3-enriched domains

**Fig 2:**
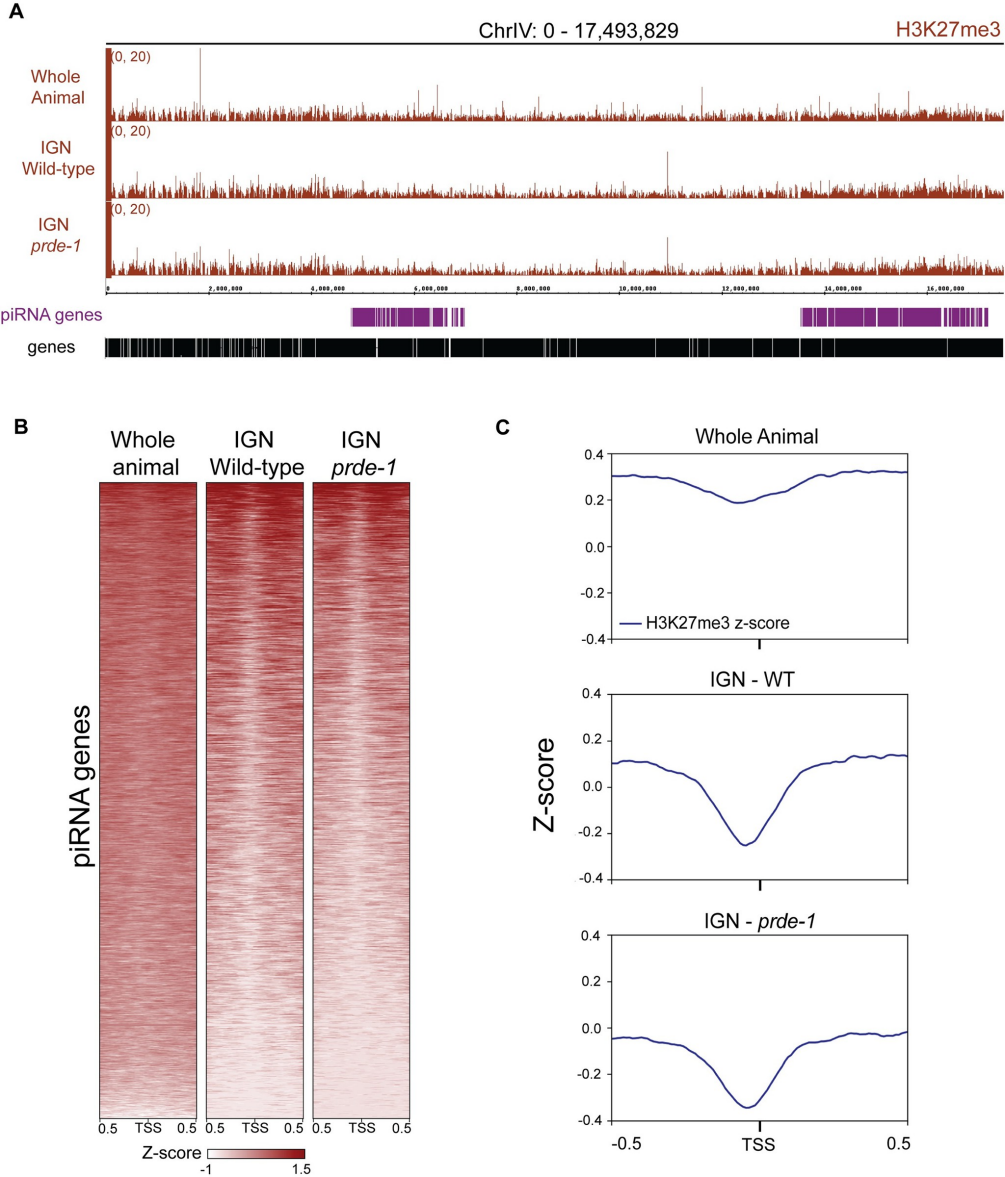
Local depletion of H3K27me3 at piRNA genes in germ nuclei. A) Genome browser view of H3K27me3 patterns across chromosome IV for whole animal, wild type IGN, and *prde-1* mutant IGN. B) Heatmap of H3K27me3 levels centered on the transcription start site (TSS) of piRNA genes within piRNA clusters for the three experiments. The H3K27me3 signal is represented as Z-score values. C) Metagene profiles (1 kb, centered on the piRNA TSS) of H3K27me3 Z-score values across piRNA genes for the three experiments.

We next investigated whether and how USTC might affect the chromatin environment at piRNA domains to support stable accumulation of USTC and TBP-1. Previous studies examining chromatin profiles from whole animal datasets found that the piRNA gene clusters are enriched for the repressive histone modification H3K27me3 [10]. However, somatic tissues were included in this analysis, while piRNA expression occurs primarily in the germline. We therefore isolated germline nuclei (IGN) [19] from wild type adults and performed chromatin immunoprecipitation followed by sequencing (ChIP-seq) for three histone modifications that significantly affect germline gene expression: the active histone modification H3K36me3 and the repressive histone modifications H3K9me3 and H3K27me3. Consistent with the whole animal analysis, the piRNA cluster is enriched for H3K27me3 in IGN (Fig 2A). Unlike the whole animal patterns, however, a strong, localized depletion of H3K27me3 was clearly apparent at individual piRNA genes in IGN, indicating that this depletion is specific to the germ line (Fig 2B). Both H3K36me3 and H3K9me3 levels are low across piRNA domains and do not change significantly in IGN compared to whole animals (S1A–S1C Fig). Both also exhibit a localized depletion at piRNA genes, although the pattern was less pronounced than H3K27me3 (S1B Fig), suggesting that the apparent depletion of H3K27me3 immediately upstream of the TSS is likely the consequence of an absence of nucleosomes in the area. These data indicate that the IGN technique provides higher resolution to capture germline-specific chromatin profiles and shows that while the broader piRNA clusters are generally enriched for H3K27me3, the immediate neighborhood surrounding individual piRNA genes is not.

To investigate whether USTC plays a role in establishing and/or maintaining the histone modification landscape of piRNA clusters, we performed IGN-ChIP-seq for H3K36me3, H3K27me3, and H3K9me3 in the *prde-1* mutant background. Overall levels of H3K27me3 across the piRNA clusters are somewhat lower in the *prde-1* mutant relative to wild type (Fig 2B), but the local depletion of H3K27me3 at individual piRNA genes persists (Fig 2C). We did not detect any significant genome-wide changes of H3K36me3 or H3K27me3 signal levels between wild type and the *prde-1* mutant (S2 Fig), although levels of H3K9me3 are very slightly increased in the *prde-1* mutant within piRNA clusters. In sum, these data show that USTC does not have a major influence on the local patterns of histone modifications at individual piRNA genes, but does promote an overall higher level of H3K27me3 across the broader piRNA clusters.

### piRNA domains exhibit low chromatin accessibility

**Fig 3:**
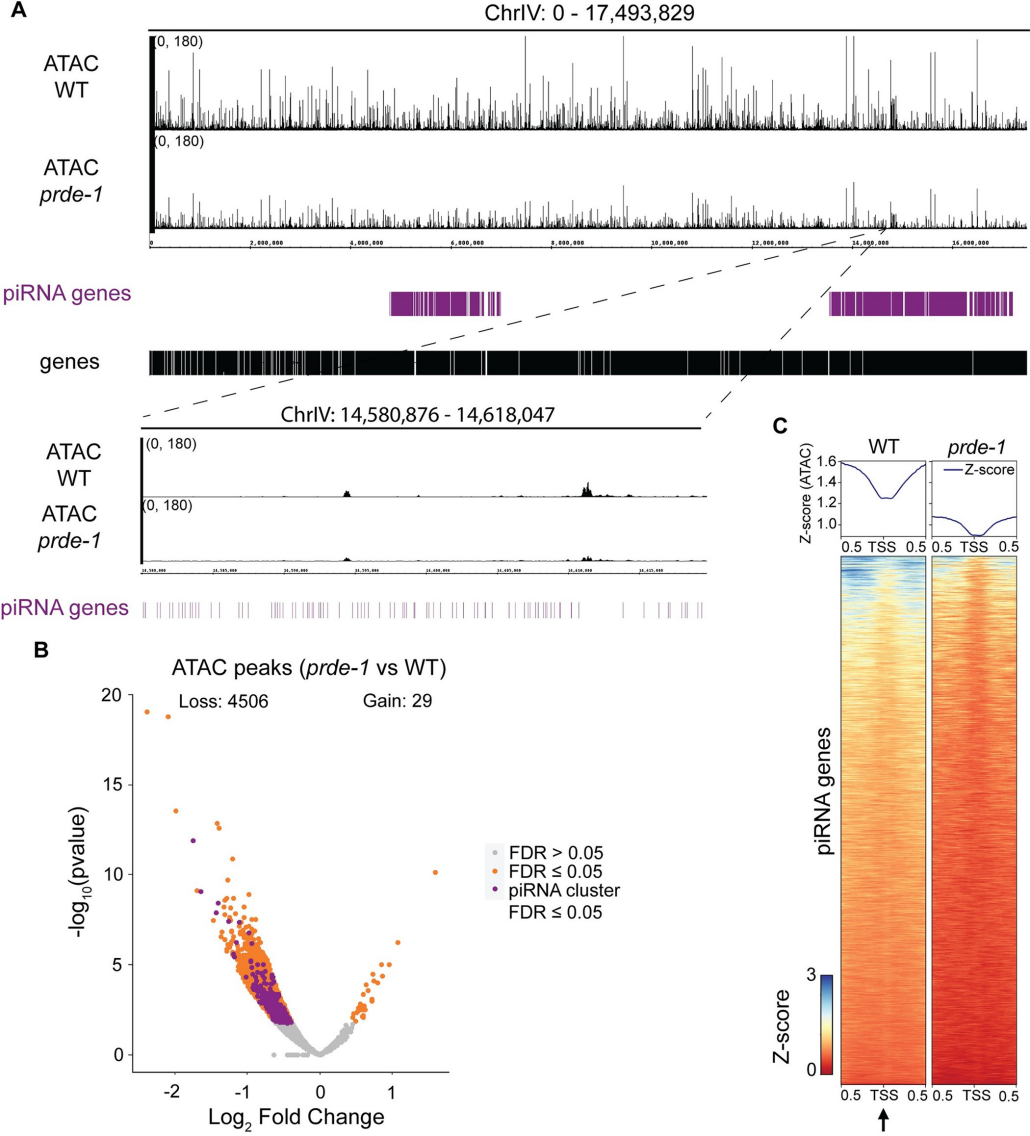
Low chromatin accessibility at piRNA clusters is further reduced in the absence of USTC. A) Genome browser view of ATAC-seq signal in wild type and *prde-1* mutant IGN across chromosome IV, with inset to show low signal at piRNA genes. B) Volcano plot representing differential chromatin accessibility peaks between *prde-1* mutants and wild type. Orange = statistically significantly different peaks (FDR ≤ 0.05). Grey = nonsignificant peaks (FDR > 0.05). Purple = differential peaks located in the piRNA domains, including both piRNA genes and coding genes, that are statistically significant (FDR ≤ 0.05). C) Metagene plots and heatmaps displaying chromatin accessibility Z-score values in wild type and *prde-1* mutants (1 kb, centered on the TSS of piRNA genes).

piRNAs are abundantly expressed in the germ line despite enrichment of the repressive mark H3K27me3 throughout the piRNA clusters. However, the localized depletion of H3K27me3 at individual piRNA genes (Fig 2B and 2C) suggests that this repressive environment might be structured in a way that promotes piRNA expression. We initially investigated overall chromatin accessibility within the piRNA gene clusters using ATAC-seq (Assay for Transposase-Accessible Chromatin using sequencing) [20] in wild type and *prde-1* mutant IGN (S3A and S3B Fig). Notably, chromatin accessibility was low overall throughout the piRNA clusters relative to the rest of the genome, and even lower in *prde-1* mutants, with the vast majority of differential peaks lost in the *prde-1* mutant (Fig 3A and 3B). Overall, most genes associated with the decreased ATAC peaks were protein-coding; piRNA genes represented only about 4% of the total (S3C Fig). Together these observations indicate that major changes in accessibility throughout the piRNA clusters largely occur at non-piRNA gene features in the region, and that piRNA domains are located in a closed chromatin environment even in the absence of USTC.

We next examined local chromatin accessibility patterns around individual piRNA genes (Fig 3A inset) in wild type and *prde-1* mutants. Overall, piRNA genes are located in regions of very low chromatin accessibility in both backgrounds (Fig 3C), although a small subset of piRNA genes is located in relatively open chromatin regions (piRNAs at top of heatmap in Fig 3C), which raises the relative accessibility of adjacent sequences in the metagene plot. Close inspection suggests a subtle increase of accessibility at the piRNA transcription start site (TSS) relative to immediately adjacent sequences (Fig 3C arrow), which was diminished in the *prde-1* mutant. Together, these results demonstrate that the piRNA clusters have low overall accessibility, and that loss of USTC function further decreases regions of open chromatin genome-wide as well as specifically within the piRNA clusters.

### Local nucleosome organization at piRNA genes is dependent on USTC

**Fig 4:**
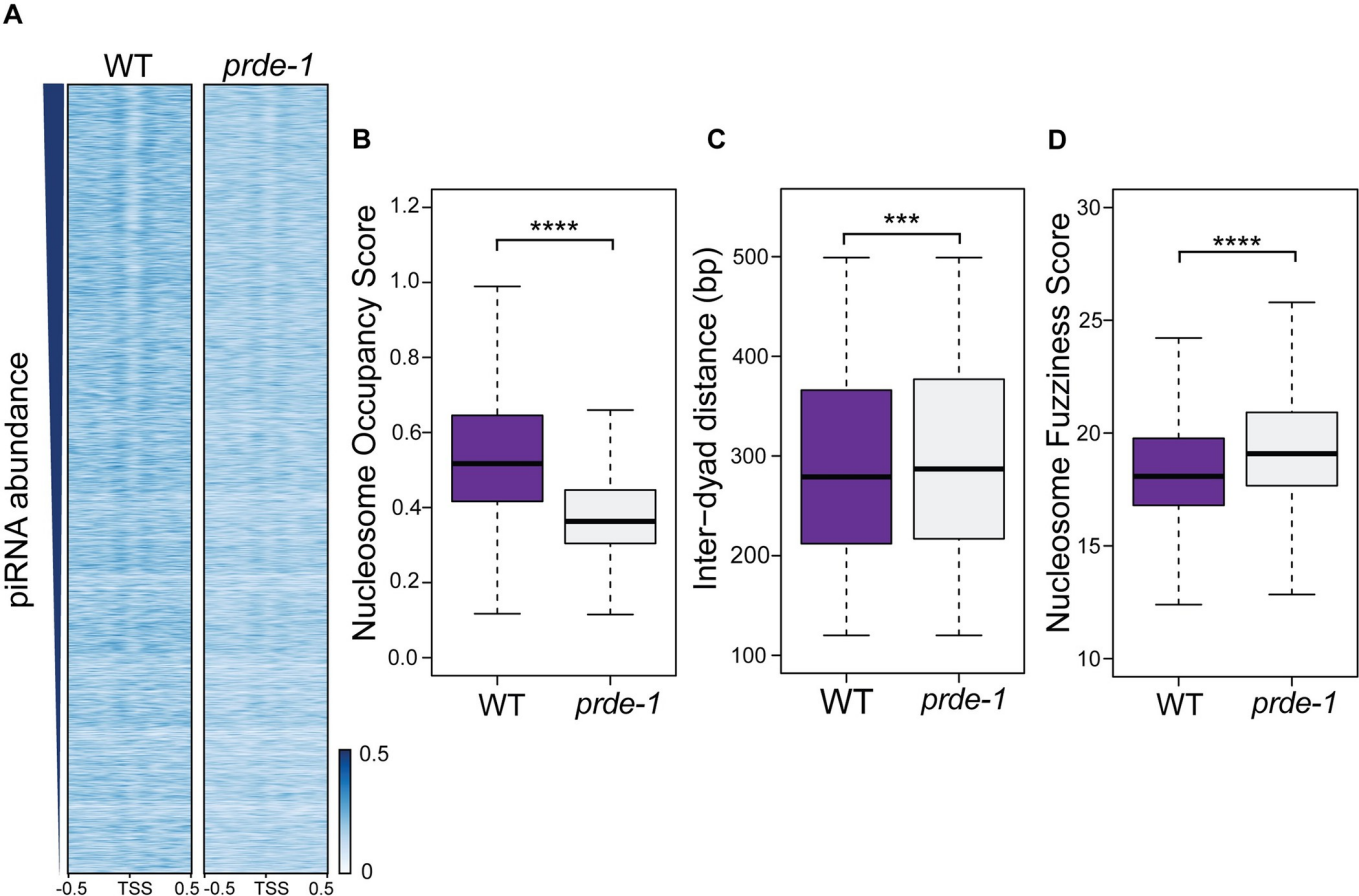
USTC promotes predicted nucleosome placement at piRNA genes. A) Metagene analysis of predicted nucleosome occupancy scores (1 kb, centered on the TSS of piRNA genes). Wild type values are in blue while *prde-1* mutant values are in green. Left plot = all piRNA genes; middle and right plot represent scores for the top 10% and bottom 10% expressed piRNA genes, respectively. B-D) Quantitative analysis of nucleosome occupancy scores, inter-dyad distance, and nucleosome fuzziness scores extracted from regions surrounding piRNA genes (±500 bp measured from the TSS). Purple represents wild type and grey represents *prde-1* mutants. Statistical significance listed for each graph was calculated with Wilcoxon test.

High levels of H3K27me3 (Fig 2) and low accessibility (Fig 3) in the piRNA clusters of wild type IGN suggest a very dense nucleosome environment, so we used the program NucleoATAC to investigate local nucleosome organization at piRNA genes at higher resolution. NucleoATAC selectively analyzes mono-nucleosome-sized fragments from ATAC-seq datasets to predict nucleosome occupancy and positioning [21]. In wild type germ cells, well-defined nucleosomes are predicted to be located immediately upstream and downstream of piRNA genes, with depletion of a nucleosome precisely at the TSS (Fig 4A). This nucleosome-free area overlaps with the site of H3K27me3 depletion (Fig 2A and 2B), as well as the subtle increase in accessibility (Fig 3C, arrow) near the TSS of piRNA genes. To determine whether this pattern correlates with piRNA expression, we stratified piRNAs by average transcript abundance from three publicly available datasets [6,7,12]. In wild type germ cells, predicted nucleosome patterns were well-defined for highly expressed piRNAs (top of heatmap), with decreasing definition of nucleosome patterns as piRNA abundance reduces (Fig 4A).

In the *prde-1* mutant, we observed decreased predicted nucleosome occupancy near the promoters of piRNA genes compared to wild type (Figs 4A and S4. Regardless of piRNA expression level, overall occupancy is lower in the *prde-1* mutant, but the individual nucleosomes predicted to flank the piRNA TSS are not as consistently positioned as in wild type (Fig 4A). Notably, for highly expressed piRNA genes, the predicted upstream nucleosome becomes much less well-defined in the *prde-1* mutant (Fig 4A, middle panel). By comparison, *prde-1*-dependent changes in predicted nucleosome patterns are not seen at genes with similar features of genomic organization and transcriptional regulation, such as genes with paused or docked RNA polymerase II [22] (S4 Fig). These data suggest that increased variability of nucleosome positioning underlies both the decreased ability to predict occupancy, as well as the overall decrease in accessibility seen in Fig 3C.

Statistical analysis of the 1-kb nucleosome environment around piRNA genes confirms a significant increase of nucleosome occupancy in the *prde-1* mutant (Fig 4B). Additionally, both inter-dyad distance, which is the distance between the centers of two neighboring nucleosomes, and nucleosome fuzziness score, which represents uncertainty in nucleosome positioning, were increased in *prde-1* mutants, suggesting that nucleosome placement is less fixed when the USTC is disrupted (Fig 4C and 4D). Finally, to independently measure the nucleosome depletion signal at piRNA genes, we calculated the Kaplan score, which predicts nucleosome placement based solely on DNA sequence, and again observed that regions immediately upstream of the TSS of piRNA genes are relatively depleted for nucleosomes (S5A Fig) [23]. Compared to germline-expressed coding genes, however, the sequence surrounding piRNA genes contributes to nucleosome occupancy to a lesser extent (S5B and S5C Fig). Together, these data are consistent with a role for USTC in organizing nucleosome patterns at piRNA genes to establish an open configuration immediately adjacent to individual piRNA loci.

### USTC promotes high histone H3 density across piRNA clusters

**Fig 5:**
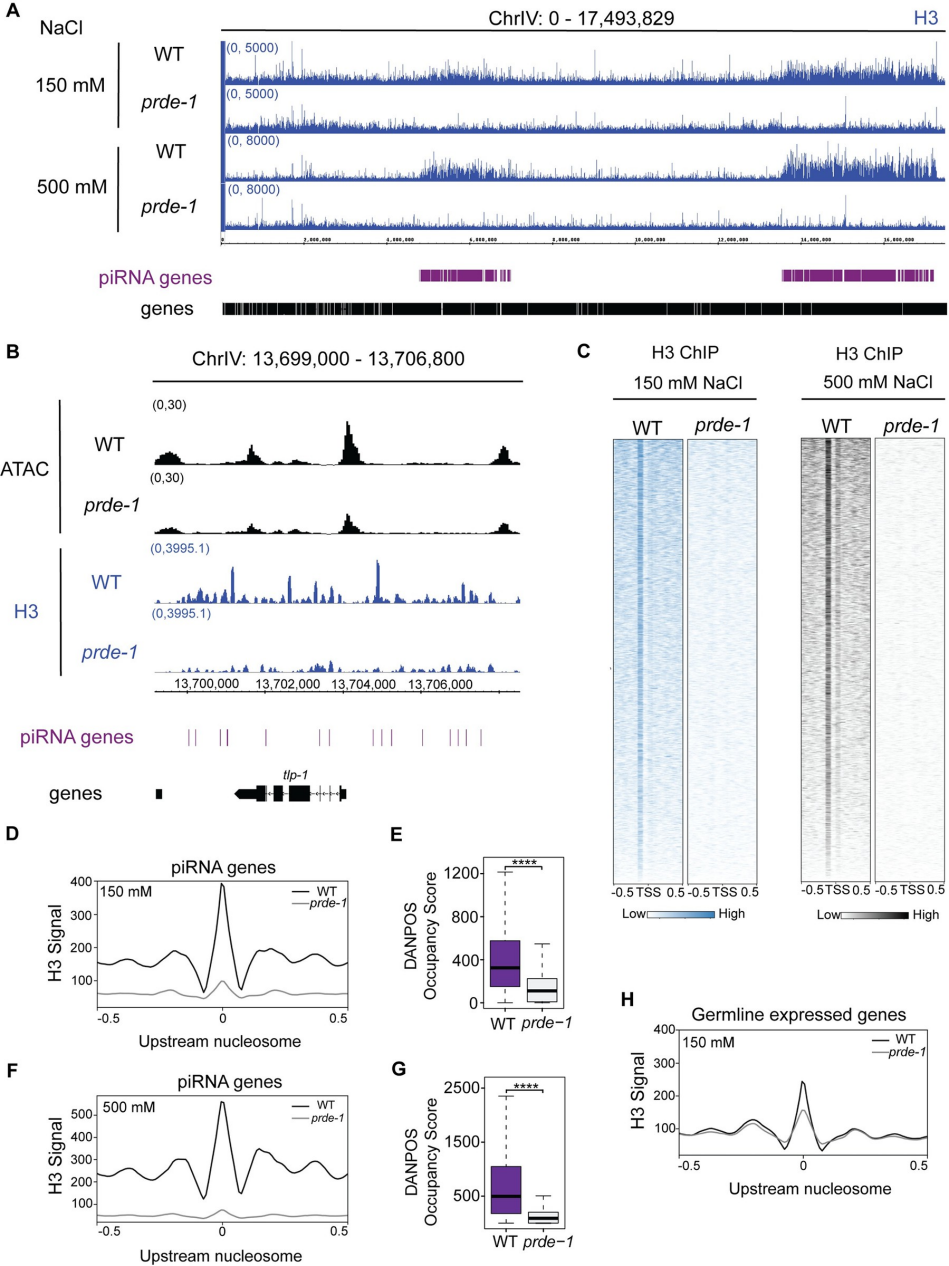
USTC maintains H3 enrichment across piRNA domains. A) Genome browser view of Native H3 ChIP-seq signal (input subtracted) at low and high salt conditions. B) Genome browser view example of inverse relationship between wild type ATAC-seq signal and wild type H3 ChIP-seq signal (150 mM salt condition). C) Heatmap representing strength of H3 signal in wild type and *prde-1* mutants at low salt (left) and high salt (right) conditions (1 kb, centered on the TSS of piRNA genes). D,F) Metagene analysis of averaged H3 signal centered at the upstream nucleosome from DANPOS at piRNA genes in low salt and high salt conditions. E,G) Quantification of occupancy for D and F, respectively. H) Metagene analysis of averaged H3 signal centered at the upstream nucleosome from DANPOS at germline-expressed genes in low salt conditions.

**Fig 6:**
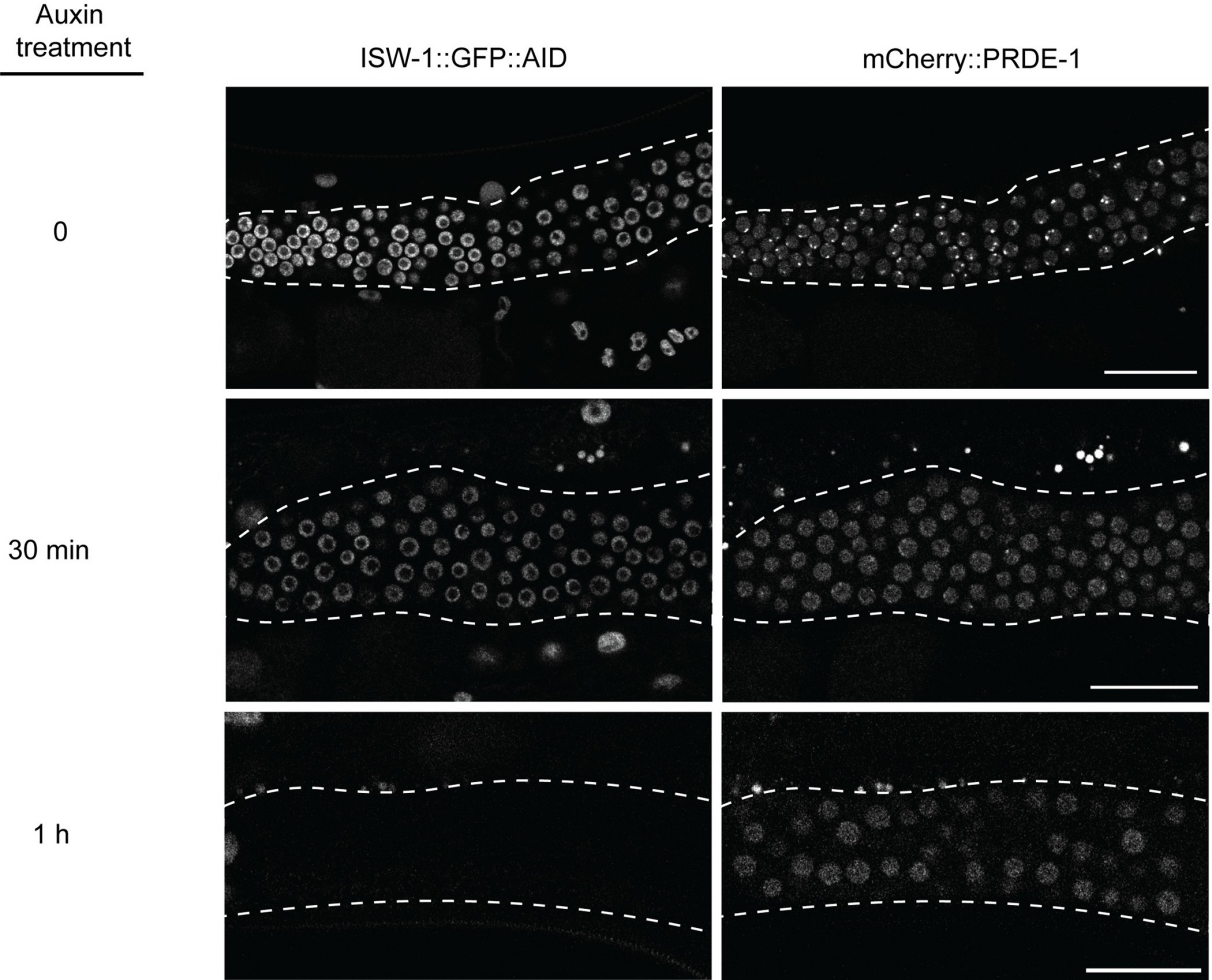
ISW-1 actively maintains USTC foci. Left panels: ISW-1::AID::GFP before (0), 30, and 60 minutes after addition of auxin. Right panels: mCherry::PRDE-1 before (0), 30, and 60 minutes after addition of auxin. Scale bar is 20 μm.

Because the NucleoATAC analysis is predictive and does not directly measure individual nucleosome placement, we independently assessed the nucleosome environment at piRNA genes by performing H3 ChIP-seq in wild type and *prde-1* mutant germline nuclei. Prior ChIP-chip data in whole worms indicated that H3 was locally depleted at piRNA genes [24] but whether this depletion occurred in the germ line was unclear. Since the ATAC-seq was performed on uncrosslinked IGN, we adapted Native ChIP-seq [25] to assess H3 location under comparable conditions (see Methods, S6A and S6B Fig). Moreover, the H3 profile has no correlation with chromatin accessibility, as expected (Figs 5B and S6C). Under standard salt conditions (150 mM), H3 levels vary across the genome, with the highest levels found across piRNA domains (Figs 5A and 6D). In *prde-1* mutants, H3 levels are significantly depleted at piRNA domains especially in comparison to the rest of the genome (Figs 5A and 6D). Notably, a strong H3 signal apparent just upstream of the TSS at piRNA genes in wild type is diminished in the *prde-1* mutant (Fig 5C–5E), suggesting that USTC maintains the upstream nucleosome at piRNA genes, consistent with the NucleoATAC analysis (Fig 4A). As a control, this effect was not seen at germline-expressed protein-coding genes (Fig 5F).

Because histone association with DNA is highly sensitive to salt concentration [26–30], we assessed whether higher salt washes altered H3 profiles, as a measure of relative nucleosome affinity for DNA. In wild type IGN, increased salt (500 mM) drastically reduced H3 association with DNA throughout the genome, with the specific exception of the two piRNA domains, which retained strikingly high levels of H3 (Figs 5A and 6D). Under this high salt condition, the precisely positioned H3 signal upstream of the piRNA TSS is even more prominent, and a second H3 signal is now apparent immediately downstream of the TSS as well (Fig 5C–5G). In the *prde-1* mutant, H3 levels were drastically depleted across the piRNA gene clusters, including complete loss of the strong H3 signal flanking the piRNA gene TSS (Fig 5C–5G). The effects of loss of *prde-1* on germline-expressed coding genes was much less dramatic than piRNAs (Fig 5H). In sum, H3 analysis in IGN independently confirms the conclusions from the ATAC-seq data and NucleoATAC analysis: that USTC both promotes high overall nucleosome density across piRNA gene clusters and establishes a local structured nucleosome environment upstream of individual piRNA genes.

### ISW-1 is required for USTC recruitment and chromatin remodeling at piRNA clusters

While USTC clearly has an important role in nucleosome organization at piRNA domains, no subunit has any apparent enzymatic activity to directly mobilize nucleosomes in chromatin. Previous work demonstrated that the ATPase chromatin remodeling enzyme ISW-1 was required for USTC recruitment to foci at piRNA domains and for piRNA expression [16]. However, this analysis was performed after long-term loss of ISW-1 activity, and could not distinguish whether ISW-1 was specifically involved in piRNA expression or whether the loss of USTC foci was an indirect consequence of global disorganization of chromatin in the absence of ISW-1.

To more precisely assess the role of ISW-1, we generated a strain in which the endogenous *isw-1* gene was edited using CRISPR-Cas9 to insert GFP and an auxin-inducible degron peptide (AID) at the carboxy-terminus. Even though ISW-1::GFP::AID expression is strong in the germ line, it does not form detectable foci (Fig 6, top middle panel). We next generated a strain that combined *isw-1*::GFP::AID with PRDE-1::mCherry and germline-expressed TIR1, which binds to AID upon auxin treatment and targets the tagged protein for degradation. We then assessed PRDE-1::mCherry focus formation at multiple times after auxin treatment. Strikingly, PRDE-1::mCherry foci largely disappear within 30 minutes, even before ISW-1 was fully depleted (Fig 6). By one hour, when ISW-1::GFP::AID is absent, PRDE-1::mCherry foci are lost, although dispersed expression can still be detected, suggesting that ISW-1 is required for USTC focus formation, but not for PRDE-1 stability. As a control, auxin treatment of PRDE-1::mCherry alone had no effect on focus formation. These data demonstrate that ISW-1 activity is continuously required to permit USTC accumulation at piRNA clusters in the adult germ line.

**Fig 7:**
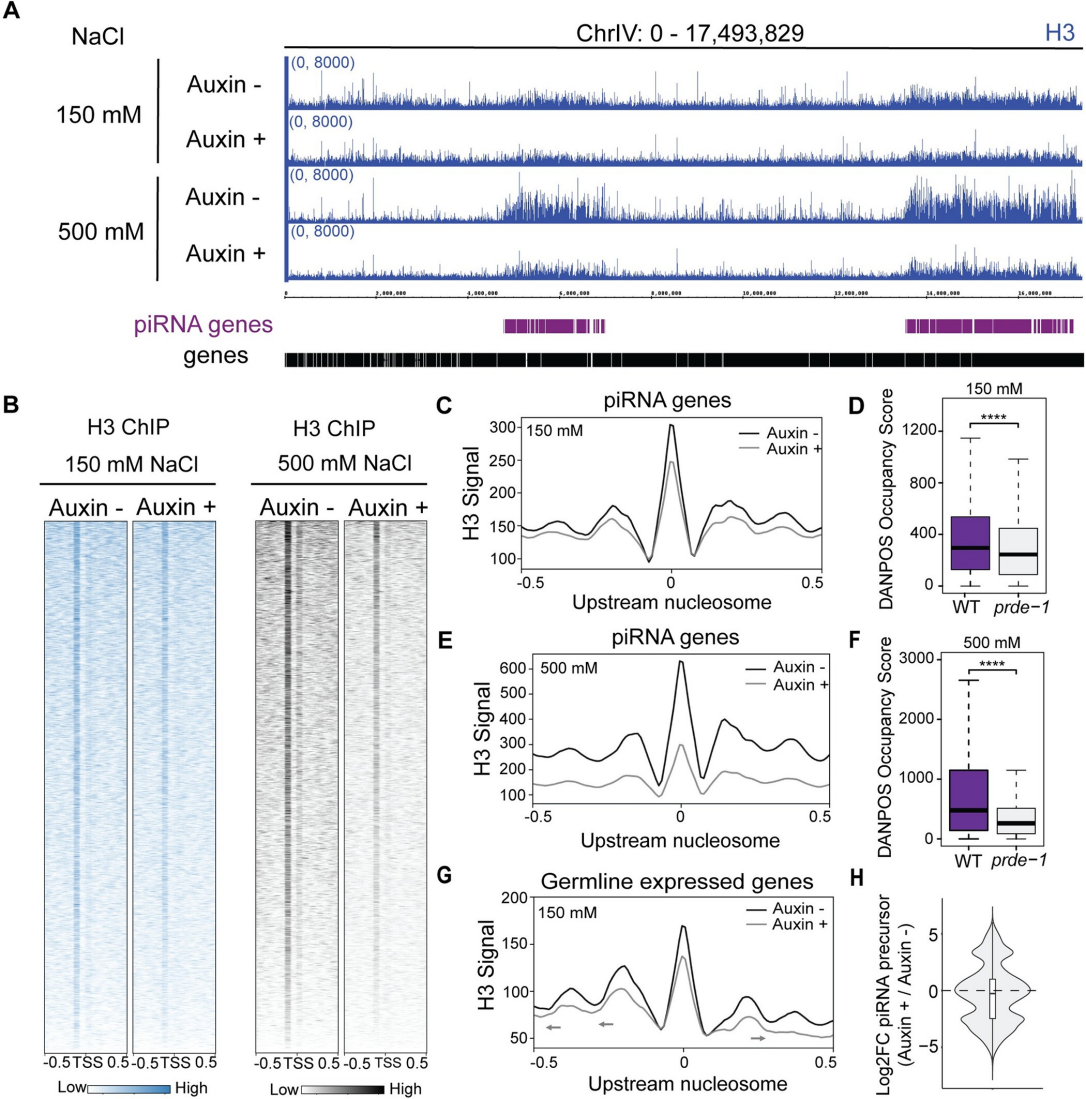
ISW-1 promotes nucleosome density across piRNA cluster. A) Genome browser view of Native H3 ChIP-seq in low and high salt conditions, before and after ISW-1 depletion by ISW-1 depletion by auxin. B) Heatmap of H3 signal centered at the TSS of piRNA genes in low and high salt conditions before and after ISW-1 depletion by auxin. C,E) Metagene analysis of averaged H3 signal centered at the called nucleosome from DANPOS at piRNA genes before (C) and after (E) ISW-1 depletion by auxin in low salt and high salt conditions. D,F) Quantification of C and E, respectively. G) Metagene analysis of H3 signal in low salt condition at germline expressed genes, centered at the upstream nucleosome. H). Quantification of piRNA precursor levels comparing ISW-1 depleted IGN to control.

We next determined whether nucleosome organization at piRNA genes is dependent on ISW-1. We performed H3 Native ChIP-seq in IGN after 3 h of auxin-induced ISW-1 degradation in standard and high salt conditions. We found a substantial loss of the characteristic high nucleosome density across piRNA clusters, especially after high salt washes (Fig 7A), indicating that ISW-1 is acutely required to maintain this density. Notably, the concentrated H3 signal immediately upstream of individual piRNAs is present after ISW-1 depletion, though slightly reduced at 150mM and considerably reduced at 500mM (Fig 7B–7D), suggesting that it persists awhile after USTC is no longer present. At piRNA genes, nucleosome positioning relative to the TSS is retained in the absence of ongoing ISW-1 activity (Fig 7C–7F), whereas at germline-expressed protein-coding genes, nucleosomes start to shift relative to the TSS (Fig 7G), consistent with the known function of ISW-1 [31]. These observations suggest that ISW-1 actively maintains nucleosome density generally throughout the piRNA domains, which promotes USTC recruitment. However, ISW-1 is less critical for the precise positioning of nucleosomes immediately adjacent to individual piRNA genes, a feature more strongly associated with USTC.

Finally, to determine whether the reduction in nucleosome density after loss of ISW-1 activity results in change to piRNA transcription, we performed IGN and isolated total RNA from ISW-1::GFP::AID animals treated with either ethanol or auxin for 3 h. We used an established strategy to preferentially clone precursor piRNAs for sequencing [6]. Indeed, we found that within 3 hours, loss of ISW-1 activity already caused a significant reduction in precursor piRNA levels (Fig 7F), indicating that piRNA transcription is indeed impaired.

## Discussion

In this report, we assess the specialized chromatin environment of the two genomic clusters of Type-I piRNA genes on Chromosome IV specifically in germline nuclei of *C*. *elegans*. We demonstrate that USTC has minimal effect on histone modification patterns in piRNA domains, but instead is primarily required for establishing a stable and organized nucleosome pattern at piRNA genes. In particular, loss of USTC disrupts the nucleosome positioning flanking the transcription start sites (TSSs) of individual piRNA genes, and the strength of this effect correlates with piRNA abundance. USTC association with the piRNA domains requires the ongoing action of the chromatin remodeling enzyme ISW-1, which maintains high nucleosome density across the domain and is required for continued production of piRNA precursor transcripts. In sum, our data demonstrate that USTC is necessary for the establishment of a locally organized chromatin environment in the germ line that facilitates piRNA expression (model; S7 Fig).

Although it is difficult to determine precise nucleosome placement using genomic assays that rely on aggregate measurements, data from several independent experiments support the conclusion that the TSS of well-expressed piRNA genes is largely accessible and flanked by two strongly associated nucleosomes precisely placed immediately upstream and downstream. First, H3K27me3 ChIP-seq data points to a local depletion of this modification immediately upstream and at the TSS (Fig 1C), which arises from either an unmodified or absent nucleosome. Second, NucleoATAC analysis of the ATAC-seq data predicts the absence of a nucleosome immediately at the TSS, with two well-positioned nucleosomes upstream and downstream (Fig 3A); this pattern correlates with piRNA abundance, suggesting that it is related to piRNA transcription. Notably, the absence of a single nucleosome would expose approximately 147 nt of DNA (not including linker sequence) [28], which is more than sufficient to encode the closely-spaced upstream regulatory motifs, promoter, and downstream motifs flanking each piRNA gene. Finally, Native ChIP-seq of H3, which does not distinguish between modified and unmodified H3, revealed an enrichment of H3 specifically upstream and downstream of, but an absence at, the TSS. Importantly, in all of the assays performed, loss of USTC function disrupts these patterns, strongly indicating that nucleosome positioning is dependent on USTC.

Our data also confirm that H3K27me3 is enriched throughout piRNA clusters in the germline, and reveal a strong local depletion of H3K27me3 at the TSSs of individual piRNA genes (Fig 1). This local H3K27me3 depletion was barely visible from whole animal data, indicating that genomic analyses in IGN greatly increase resolution and specificity. However, USTC does not seem to be directly involved in establishing these H3K27me3 patterns. Disrupting USTC function has no effect on the depletion of H3K27me3 at piRNA TSSs and leads to only a mild reduction in H3K27me3 level across the piRNA clusters, likely as a secondary consequence of decreased nucleosome density. Thus, H3K27me3 deposition is likely regulated by an independent mechanism. Indeed, the *C*. *elegans* orthologs of PRC2, a histone methyltransferase complex that methylates H3K27, as well as UAD-2, an H3K27me3 reader, are necessary for USTC binding at piRNA clusters [16]. Together, these observations suggest that deposition of H3K27me3 by PRC2 occurs independently of, and is likely important for, USTC binding.

The use of heterochromatin to mark piRNA clusters and recruit specific regulatory factors for piRNA gene expression is not unique to *C*. *elegans*. In Drosophila, piRNA precursors located in dual-strand clusters are dependent on co-occupancy of H3K27me3 and H3K9me3 at the promoter, which leads to the recruitment of the Rhino protein and subsequent transcription of the precursor RNA [32–36]. Thus, H3K27me3 and USTC in *C*. *elegans* may be analogous to H3K9me3/H3K27me3 and Rhino in Drosophila. This similarity between species occurs despite profound differences in the two systems: Drosophila piRNAs largely encode repetitive, transposon-rich sequences, and are produced as large precursor RNAs that are subsequently cleaved into many piRNAs, while in *C*. *elegans*, piRNAs are incredibly sequence diverse and are independently transcribed from tiny individual genes [3]. The mechanistic reasons why a heterochromatin-like state is required at piRNA genomic loci is unclear, but for *C*. *elegans*, it is possible that a dense nucleosomal neighborhood surrounding the short stretches of open chromatin at piRNA genes restricts RNA polymerase II processivity and facilitates the production of short precursor piRNAs.

Overall, our data are consistent with a model in which both the nucleosome remodeler ISW-1 and H3K27me3 create a specialized, closed chromatin environment that marks the piRNA genomic domains. In Neurospora, ISWI is required for PRC2 to be recruited to chromatin and establish proper H3K27me3 levels [37]. Potentially, a similar functional relationship between ISW-1 and PRC2 (MES-2/MES-3/MES-6) could exist in the *C*. *elegans* germ line. We determined that ISW-1 is actively required to maintain USTC foci by promoting nucleosome density at the piRNA genomic domains, but whether ongoing PRC2 activity is also required is unknown. In this model, USTC binding at the Ruby motif then allows for precise positioning of nucleosome upstream and downstream of piRNA genes, leaving the piRNA genes accessible for TBP-1 and RNA polymerase II. Notably, the two USTC subunits SNPC-4 and TOFU-4 have SANT domains, which can directly interact with histones. Thus, one possibility is that USTC acts similar to a pioneer transcription factor by binding directly to nucleosomes flanking the Ruby motif and stably positioning them to facilitate TBP-1 binding, RNA polymerase II engagement, and transcription initiation. However, we currently cannot rule out that the nucleosome positioning seen at individual piRNA genes is a consequence of active RNA polymerase II, and that the role of USTC is to recruit RNA polymerase II via TBP-1, which then initiates transcription, leading to nucleosome displacement at the promoter and stabilization of upstream and downstream nucleosomes. Structural and biochemical studies will be required to better understand the precise mechanisms by which USTC coordinates piRNA expression in the germ line of *C*. *elegans*.

## Materials and methods

### *C*. *elegans* strains

Strains were maintained at 20°C on NGM plates seeded with OP50 [38]. The JDW223, YL700 and YL704 strains were maintained at 22°C on NGM. VC2010 was used as the wild type strain, and the SX2499 *prde-1(mj207)* strain was used as the *prde-1* null mutant strain [13]. Importantly, because of the progressive sterility of SX2499, we backcrossed this strain to N2 twice and performed all genomic experiments within the minimal generation number necessary for worm growth. All strains used in this study are listed in S1 Table.

### CRISPR/Cas9 genome editing

For the generation of the ISW-1::GFP::AID knock-in, the C-terminus of *isw-1* was targeted with crRNA, 5’-GCTTTGACTTTCTTAGCAGTTGG-3’. The GFP sequence was obtained from [39]. The repair template containing GFP::AID was synthesized as a Gblock by IDT with 120 bp homology arms (S8 Fig). Then the template was PCR amplified with or without the homology arms were gel purified by Zymoclean Gel DNA Recovery and then mixed at 1:1 [40]. The donor mixture was then melted [95°C 2:00 min, 85°C 10 s, 75°C 10 s, 65°C 10 s, 55°C 1:00 min, 45°C 30 s, 35°C 10 s, 25°C 10 s, 4°C hold] to generate hybrid DNA donors [41]. Young adult VC2010 animals were injected with Cas9 protein (30 pmol, IDT), Cas9 tracrRNA (90 pmol, IDT), crRNA (95 pmol, IDT), repair template (900 fmol), and pRF4 (177 fmol) as previously described [42]. F1 progeny (rollers and non-rollers) were screened for GFP expression. The resulting strain was backcrossed 3X.

### Auxin treatment

Worms were synchronized by bleaching gravid worms and hatched overnight in M9 for 16–24 h. Then 300 L1s were plated onto NGM plates. After 45 h, YL704 was transferred onto NGM plates containing either 1 mM auxin or 1mM ethanol for 30 min or 1 h.

### Microscopy and image acquisition

Worms were bleached for synchronization. Strains were imaged after 49 h for YL689, OP746 or 47 h for YL704. Worms were picked onto an unseeded NGM plate for 5 minutes to allow worms to remove adherent bacteria. The worms were mounted onto 5% agarose pads with 10 mM Tetramisole (Sigma) and No.1.5 coverslip (Corning). Worms were imaged with Plan-Apochromat 63X 1.4 NA oil immersion objective on Zeiss 980 laser scanning confocal microscope with Airyscan2.

GFP and mCherry fluorescence were excited with the 488 nm (0.57% laser power) and 561 nm (0.59% laser power), respectively. Images acquired in Super Resolution mode (SR) at zoom 1x, with a frame size of 4096 x 4096 px, a pixel time of 2.65 us, in 8-bit, and in bidirectional mode. The images were processed post-acquisition using the “auto” mode and saved in CZI format.

### Isolation of germline nuclei

Isolated germline nuclei (IGN) were harvested as in [19] with minor modifications. Worms were harvested at 49–52 hours for wild type and 56–59 hours for *prde-1(-)*, when most animals had 4–6 embryos. For auxin-mediated knockdown, L1 worms were grown on peptone enriched plates for 45 hours, then moved to peptone enriched plates with 1 mM auxin or ETOH plates for 3 hours. Approximately 1.2 million worms were used per isolation. Worms were washed with 1x M9 in sets of 6 plates into a 50 mL falcon tube, washed twice with M9, floated on sucrose, and washed again 3x in M9.

#### IGN for ChIP-seq

At the adult stage, worms were crosslinked for 30 min in 2% formaldehyde diluted in M9. Formaldehyde was quenched by a 1 M Tris (pH 7.5) wash and washed twice with M9. Then the worms were washed with 10 mL chilled Nuclei Purification Buffer (NPB) (50 mM HEPES pH 7.5, 40 mM NaCl, 90 mM KCl, 2 mM EDTA, 0.5 mM EGTA, 0.1% Tween 20, 0.2 mM DTT, 0.5 mM PMSF, 0.5 mM spermidine, 0.25 mM spermine, and 1x Complete Protease Inhibitor Cocktail). Worms were resuspended in 6 mL of NPB and transferred into prechilled 7 mL glass Dounce homogenizers (Wheaton cat. no. 06435A). Fifteen loose strokes were followed by 23 tight strokes with a quarter turn between each Dounce. The worms were transferred into chilled 50 mL Falcon tubes, and NPB was added to 10 mL. The tubes were vortexed on medium-high speed for 30 s, followed by 5 min on ice. The vortex and ice incubation were repeated. Worm debris was filtered with four 30 μm strainers (MACS cat. no. 130-098-458) and seven 20 μm strainers (Pluriselect cat. no. 43-50020-03). Nuclei were spun at 3100 rpm for 6 min at 4°C. The supernatant was removed, and the nuclei resuspended in 1 mL NPB and transferred to a nonstick 1.5 mL tube (Ambion). A 5 μl aliquot was removed, incubated with DAPI for 10 min, and counted using a hemacytometer (Hausser Scientific). Finally, the remaining nuclei were spun at 4°C at 4000 rpm for 5 min, the supernatant was removed, and the pellet was flash frozen in liquid nitrogen. The nuclei were stored at −80°C until sonication.

#### IGN for Native ChIP-seq and ATAC-seq

Approximately 1.2 million worms were used per isolation for Native ChIP-seq, and 400,000 worms were used per isolation for ATAC-seq. After the final wash with M9, worms were washed with 10 mL NPB. The supernatant was removed and the worms were resuspended in 6 mL of M9 and transferred to a pre-chilled 7 mL glass Dounce homogenizer. All of the Dounce and following steps were performed as for ChIP-seq except for the number of strainers. Worm debris was strained with three 30 μm strainers and six 20 μm strainers. The samples were resuspended in 1 mL NPB and left on ice until starting the desired genomic assay.

#### IGN for small RNA-seq

Approximately 400,000 worms were used per isolation. Worms were washed with M9 three times, then incubated with M9 for 45 min– 1h with shaking to reduce intestinal bacteria. After incubation, worms were washed with 10 mL NPB. The supernatant was removed, and the worms were resuspended in 6mL of M9 and transferred to a pre-chilled 7mL glass Dounce homogenizer that was pre-washed once with RNase Away (Thermo Scientific) and then rinsed twice with RNase Free water. Protector RNase Inhibitor (100 μL, Sigma) was added per homogenizer. All of the Douncing and following steps were performed as for ATAC-seq. The samples were resuspended in 1 mL NPB with 25 μL of Protector RNase Inhibitor. Then the nuclei were spun at 4°C at 4000 rpm for 5 min, the supernatant was removed, and resuspend in 500 μL of Trizol (Invitrogen). The solution was flash frozen in liquid nitrogen and −80°C until treatment.

### ChIP-seq

For each sample, ~20 million IGN were used. ChIP was performed as described previously [19]. For each IP, 5 μg of H3K27me3 antibody (Active Motif cat. no. 61018), 5 μg of H3K36me3 antibody (Active Motif cat. no. 61022), or 5 μg of anti-H3K9me3 (Abcam, #8898). Each IP was spiked with 90 ng of *Drosophila* spike-in chromatin (Active Motif cat. no. 53083) and 0.5 μg of *Drosophila*-specific H2Av antibody was added to each sample.

For Native ChIP-seq, ~12 million nuclei were used per sample. We adapted the protocol as described in [25]. Briefly, nuclei were washed in 1x PBS + 1x Complete Protease Inhibitor Cocktail EDTA-free and pelleted at 2000g for 10 min. Nuclei were resuspended with 300 μL of Mnase buffer (50 mM HEPES pH 7.5, 110 mM NaCl, 40 mM KCl, 2 mM MgCl2, 1 mM CaCl2). Then 50 μL were aliquoted into 1.5 ml LoBind tubes(Eppendorf cat. no. 022431021). For each aliquot, 50 μL of Lysis Buffer 1 (1 mM Tris–HCl pH7.5, 1 mM PMSF, 1 mM DTT, 0.1% Tween 20, 0.2% NP-40, 0.01% digitonin) was added to each tube and incubated for 10 minutes. After the 10-minute incubation, 10 U of Mnase (Sigma cat no. 10107921001) was added to each aliquot. Then 100 μL of Mnase buffer was added, mixed by pipetting, and placed at 37°C for 4 min and 30 sec. Mnase digestion was stopped by adding 4 μL of 0.5M EDTA and then spun for 17000g for 10 min at 4°C. The supernatant was transferred to a fresh 1.5 mL LoBind tube. The pellet was resuspended in 250 μl of Lysis Buffer 2 (1 mM Tris–HCl pH7.5,1 mM PMSF, 1 mM DTT, 0.1% Tween 20, 1% Triton X-100 + 1x Complete Protease Inhibitor Cocktail) and then incubated for 2 hours while rotating at 4°C. Chromatin was pre-cleared with 40 μL of Dynabeads protein G (Invitrogen cat no. 10003D) for 1 hour with rotation at 4°C. For input, 10% of lysate was set aside. For IP set up, 5 μg of H3 antibody (Abcam cat. no. ab1791) was added. Each IP was spiked with 10 ng of *Drosophila* spike-in chromatin (Active Motif cat. no. 53083) and 0.5 μg of *Drosophila*-specific H2Av antibody was added to each sample and incubated overnight with rotation at 4°C. The following day, 40 μL of pre-blocked beads were added and incubated with rotation at 4°C for 2 hours. For the high salt wash, the ChIP samples with beads were washed 2 times with FA-150mM NaCl (50 mM HEPES pH 7.5, 1 mM EDTA pH 8.0, 1% Triton X-100, 0.1% Na deoxycholate, 150 mM NaCl and 1x cOmplete proteinase inhibitor cocktail EDTA-free (Roche)) and 2 times with FA-500mM NaCl (50 mM HEPES pH 7.5, 1 mM EDTA pH 8.0, 1% Triton X-100, 0.1% Na deoxycholate, 500 mM NaCl and 1x cOmplete proteinase inhibitor cocktail EDTA-free (Roche)). For the low salt wash, the ChIP samples were washed 2 times with FA-75 mM NaCl (50 mM HEPES pH 7.5, 1 mM EDTA pH 8.0, 1% Triton X-100, 0.1% Na deoxycholate, 75 mM NaCl and 1x cOmplete proteinase inhibitor cocktail EDTA-free (Roche)) and 2 times with FA-150 mM NaCl (50 mM HEPES pH 7.5, 1 mM EDTA pH 8.0, 1% Triton X-100, 0.1% Na deoxycholate, 150 mM NaCl and 1x cOmplete proteinase inhibitor cocktail EDTA-free (Roche)). The protein-DNA complex was eluted with 150 μL of elution buffer (100 mM NaHCO3, 0.2% SDS, 5 mM DTT, 1x TE) and incubated in the thermomixer at 65°C for 15 min at 800 rpm. The elution step was repeated once more, and the supernatants were combined. For input, samples were brought up to 300 μl with 1x TE. For both input and ChIP samples, 10 μL of RNAse A (10 mg/mL) was added and incubated in a thermomixer at 37°C for 15 min at 800 rpm. Input and ChIP samples were digested with 3 μl proteinase K (20 mg/ml) at 55°C overnight.

For sequencing preparation, the input and ChIP samples were purified with Zymo ChIP DNA Clean & Concentrator kit (Zymo Research #D5205) according to the manufacturer’s instructions. The Yale Center for Genome Analysis (YCGA) prepared the library and performed sequencing. DNA integrity and fragment size were confirmed on a Bioanalyzer. Samples were sequenced on an Illumina NovaSeq using 100 bp paired-end sequencing.

### ATAC-seq

For each sample, approximately 50,000 nuclei were used. The ATAC library was prepared as previously described [43]. Nuclei were washed with ATAC-Resuspension Lysis Buffer (10 mM Tris-HCl pH 7.4, 10 mM NaCl, 3 mM MgCl_2_, 0.1% Tween 20, and 0.01% Digitonin) then spun at 2000 rpm for 10 min. Nuclei were then washed with ATAC-Resuspension Wash Buffer (10 mM Tris-HCl pH 7.4, 10 mM NaCl, 3 mM MgCl_2_, and 0.1% Tween 20). The nuclei pellet was resuspended in 50 μl transposition mixture (2X Tagment DNA Buffer, 1X PBS, 0.01% Digitonin, 0.1% Tween-20, 100 mM Tn5 Transposase) and incubated at 37°C for 30 min, shaking at 1000 rpm. The reaction was purified with Zymo DNA Clean and Concentrator-5 Kit (cat# D4014) according to the manufacturer’s instructions. DNA was pre-amplified with 2× NEBNext Master Mix (NEB M0541S). Library amplification was assessed by qPCR on Applied Biosystems QuantStudio 3 real-time PCR system. The library was purified with Zymo ChIP DNA Clean & Concentrator kit (Zymo Research #D4014) according to the manufacturer’s instructions. The Yale Center for Genome Analysis (YCGA) prepared the library and performed sequencing. DNA integrity and fragment size were confirmed on a Bioanalyzer. Samples were sequenced on an Illumina NovaSeq using 100 bp paired-end sequencing.

### Small RNA-seq

Samples were subjected to three freeze/thaw cycles (thawed for 2 min at 37°C/flash frozen for 1 min). Samples were centrifuged for 5 min at 14800 g, and supernatants transferred to a new tube and mixed 1:1 with cold 100% ethanol. RNA isolation was performed with Direct-zol RNA microprep kit (Zymo Research #R2062). RNA was eluted with 15 μL of nuclease-free water (Zymo Research) and treated with 2 μL Quick CIP (NEB M0525S) in rCutSmart Buffer (NEB B6004S) in a total reaction volume of 20 μL at 37°C for 90 min. CIP-treated RNA samples were heat-inactivated for 2 min at 80°C, suspended in 100 μL Trizol (Invitrogen) and purified with Direct-zol RNA microprep kit (Zymo Research #R2062). The RNA was then treated with 1.5 μL RppH (NEB M0356S) in a total reaction volume of 20 μL for 1 h at 37°C, resuspended in 100 μL Trizol (Invitrogen) and purified with Direct-zol RNA microprep kit (Zymo Research #R2062). The Yale Center for Genome Analysis (YCGA) prepared the small RNA library with the QiaSeq miRNA library prep kit (Qiagen) and performed sequencing on an Illumina NovaSeq using 75 bp paired-end sequencing. The RNA quality and fragment size distribution were confirmed with a Bioanalyzer.

### Computational analysis of genomic data

#### ChIP-seq processing

The quality of raw ChIP-seq data was assessed using FastQC [44], then mapped to ce11 with bowtie2 with default paired-end parameters (Langmead & Salzberg, 2012). To identify and filter for spike-in reads, raw ChIP-seq data were mapped to dm6 with bowtie2 with default settings. Whole animal histone ChIP-seq data were analyzed from available data: GSM3141776 and GSM3141788 [45], and GSM2515718 [46]. These data were first mapped with bowtie2. The aligned reads were further processed by samtools to retain high quality reads (Q ≥ 30), and duplicate reads were removed by Picard MarkDuplicates. To minimize replication bias, bam files were downsampled to the size of the replicate with smaller library size, and then merged by samtools. For whole animal datasets, bam files were converted to bigwig tracks using deeptools bamCoverage -bs 50 –normalizeUsing CPM [47]. For spike-in normalization, the merged bam files were processed in R using ChIPSeqSpike package [48]. Bigwig files were converted to z-score using custom R scripts. Peaks were called with MACS2 [49] with the parameters −q 0.05 –extsize 200. For Native ChIP-seq, the merged bam files were GC corrected and then mononucleosome sized fragments were selected (140–200 bp). The filtered bam files were converted to bigwig tracks and nucleosomes were identified by DANPOS3 [50], using input as control with parameters using input as control with parameters -m 1 –extend 70 -c 93260000 -u 0 -z 1 -a 1 -e 1 -b. The upstream nucleosome was obtained by BEDTools [51] intersect within 200 bp upstream of the TSS from published piRNA and germline expressed genes [10,19]. Metagene plots and heatmaps were generated using deepTools. Custom R script was used to generate scatterplots. Metagene plots and heatmaps were generated using deepTools.

#### ATAC-seq processing

The quality of ATAC datasets were assessed with FastQC. Adapters were trimmed with cutadapt [52] with the following parameters -a CTGTCTCTTATACACATCT. Files were aligned to ce11 with bowtie2 with the follow settings -X 2000 –no-mixed–no-discordant–very-sensitive. Bam files were processed for mapped quality Q ≥ 10, mitochondria reads were removed with samtools and duplicates were removed with Picard MarkDuplicates. Replicates were downsampled to minimize replication bias and then merged. Reads were then filtered for fragment size less than 100 bp. Reads mapped to the + strand were offset by +4 bp and reads mapped to the–strand were offset by -5 bp. Bam files were converted to bigwigs by normalizing to RPM and converted to z-score using custom R scripts. Peaks were called using Genrich (https://github.com/jsh58/Genrich) with the following parameters -j -y -v. HOMER findMotifsGenome.pl with the parameter size -given was used to determine motif enrichment from ATAC peaks. The HOMER function annotatePeaks.pl was used to determine genes associated with differential peak analysis. Metagene and heatmaps were generated using deepTools.

#### Nucleosome occupancy

Merged bam files were used as inputs for NucleoATAC [21] using default settings. The occ.bedgraph files were used to visualize nucleosome occupancy. The NucleoATACR package was used to retrieve nucleosome occupancy, inter-dyad distance, and fuzziness scores, which were further analyzed using custom R scripts. Statistical significance was determined by Wilcoxon test.

#### Differential peak analysis

To identify differential peaks in the ATAC dataset, the Bioconductor package DiffBind [53] was used with default settings. Filtered bam files and Genrich generated consensus peaks were used as inputs. Volcano plot was generated using custom R scripts.

#### piRNA expression analysis

piRNA expression data was obtained by publicly available data [6,7,12]. Reads were trimmed for adapters and aligned to ce11 with bowtie2. Reads matching piRNA annotations were matched and normalized with DESeq2. Custom R scripts were used to generate decile rank of piRNA abundance.

#### piRNA precursor analysis

Adapters were trimmed with cutadapt [52] from raw reads, and then reads were size selected for > 17 nt and by the following parameters ‘-m 26 -a GTTCAGAGTTCTACAGTCCGACGATC -a AACTGTAGGCACCATCAAT -a AGATCGGAAGAG.’ The quality was verified with FastQC [44], then mapped to ce11 with bowtie (http://bowtie-bio.sourceforge.net) with the following parameters ‘-m 1 -v 0—trim5 4—trim3 4’. The sam files were converted to bam file via samtools. To obtain piRNA precursor read counts, the annotated 5′ ends of mature piRNAs bed file was extended 2 nt then converted to saf file. Only reads that mapped to 2 nt upstream of annotated piRNAs were identified as piRNA precursors via Subread featureCounts with the following parameters ‘featureCounts -s 1 -M—read2pos 5’. The library was normalized reads per million via custom R scripts. To calculate the log2 fold change, a pseudocount of 1 was added to avoid zero counts and calculated for loci with more than 5 reads per million via custom R script.

## Supporting information

S1 Fig piRNA genes have relatively low levels of H3K36me3 and H3K9me3 in germ nuclei. A) Heatmap of H3K36me3 and H3K9me3 levels of piRNA genes in piRNA clusters (1kb, centered on the piRNA TSS). The signal is represented as Z-scores. B) Metagene profile of H3K9me3 Z-score values across piRNA genes (1kb, centered on the piRNA TSS) located within H3K9me3 peaks. C) Metagene profile of H3K36me3 Z-score values across piRNA genes (1kb, centered on the piRNA TSS).
(PDF)

S2 Fig Subtle changes in repressive histone signals at piRNA clusters. Histone modification violin plots for: A) H3K27me3 B) H3K9me3 C) H3K36me3. Violin plots showing log2fold change of histone modification signal at their respective peaks in *prde-1* mutants relative to wild type. “Outside” refers to genomic regions on ChrIV that exclude piRNA gene cluster regions. “Whole genome” refers to genomic locations from all five autosomes and the X chromosome.
(PDF)

S3 Fig Differential chromatin accessibility peak loss is associated with protein-coding genes. A) Principal component analysis of ATAC-seq replicates. Wild type—blue and *prde-1* mutants—pink. B) Correlation scatterplot between wild type and *prde-1* Z-score values, in 100bp bins. C) The y-axis of the stacked barplot represents percentage of associated genes from peaks significantly lost in the *prde-1* mutant.
(PDF)

S4 Fig Loss of USTC does not affect nucleosome occupancy at RNA Pol II paused and docked genes. Metagene analysis of nucleosome occupancy scores (1kb, centered on the TSS of piRNA genes). Wild type values are in blue while *prde-1* mutant values are in green for piRNA genes (left panel), RNA Pol II paused genes (middle panel), and RNA Pol II docked genes (right panel) [22].
(PDF)

S5 Fig Predicted nucleosome score via Kaplan score depicts lack of nucleosome signal at piRNA genes. A) Heatmap representing Kaplan score centered at the TSS of piRNA genes. B) Metagene analysis of Kaplan score at piRNA genes. C) Kaplan score metagene analysis at germline-expressed genes centered at the TSS.
(PDF)

S6 Fig High density of H3 signal is retained at ChrIV. A-B) Genome browser shot within (A) and outside (B) the piRNA cluster under low salt conditions. C) Pearson correlation between ATAC-seq and low salt Native H3 ChIP-seq datasets, in 50bp bin. D) Genome browser shot of each individual chromosome of H3 signal in wild type and *prde-1* mutants at low and high salt conditions. F) Quantification of upstream nucleosome occupancy score between WT and *prde-1* mutant from DANPOS in low and high salt conditions.
(PDF)

S7 Fig Summary of piRNA biogenesis. A) Nucleosome occupancy score at piRNA loci generated by NucleoATAC. B) H3 native ChIP-seq analysis at the piRNA locus in WT and *prde-1* mutants. C) H3 native ChIP-seq after 3 h of ISW-1 KD vs ETOH control at piRNA locus. A-C) The magenta line represents 5’ end of RUBY motif and the 5’ U represents piRNA genes. D) Model of piRNA biogenesis. In the absence of ISW-1 and USTC, nucleosome positioning is highly variable, and regulatory sequences cannot be accessed. ISW-1 promotes nucleosome density and promotes USTC binding at the Ruby motif upstream of the piRNA TSS. Recruitment of TBP-1 by USTC then facilitates association of RNA polymerase II.
(PDF)

S8 Fig Template sequences used in this study. A) Gblock sequence used for CRISPR-Cas9. B) crRNA sequence used for CRISPR-Cas9. C) All other primer sequences used in this study.
(PDF)

S1 Table Strains used in this study. (DOCX)
