# Supplementary figures and images for: The Upstream Sequence Transcription Complex dictates nucleosome positioning and promoter accessibility at piRNA genes in the C. elegans germ line
Source: PLoS Genet. 2024 Jul 10;20(7):e1011345. doi: 10.1371/journal.pgen.1011345 (PMC11262695; doi:10.1371/journal.pgen.1011345)

Supplemental Figure 1.

A

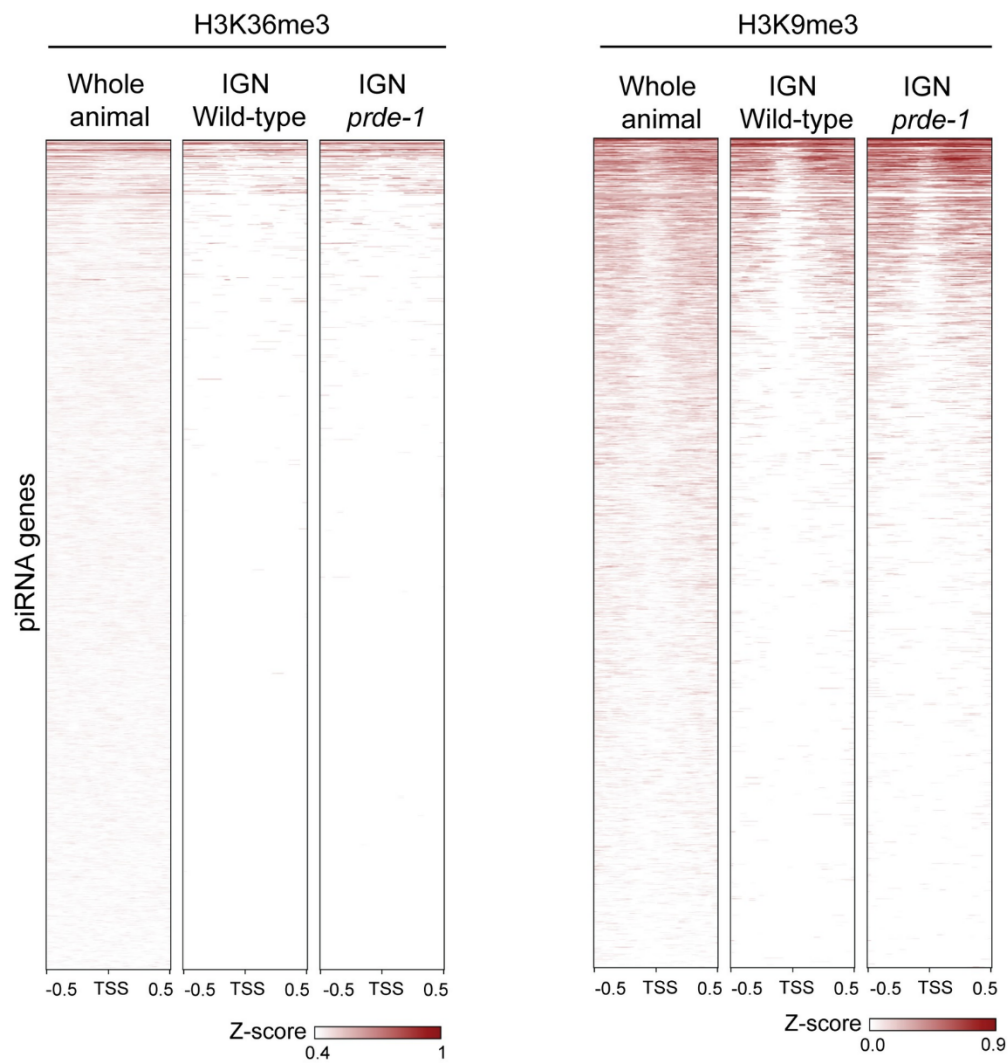

B

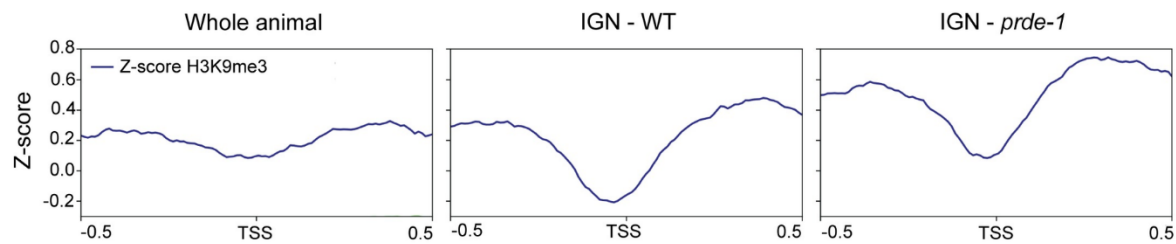

C

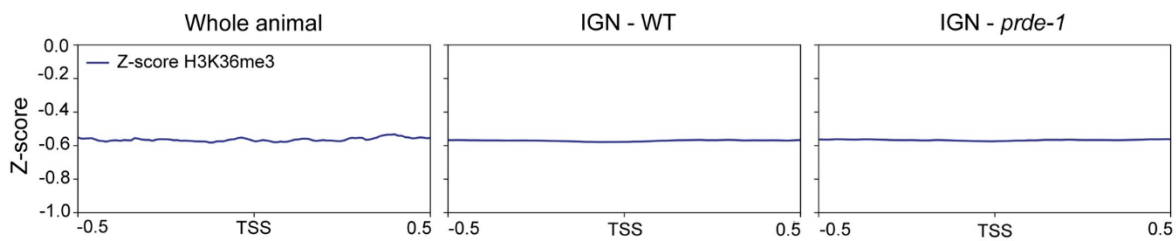

Supplement: S1 Fig — A) Heatmap of H3K36me3 and H3K9me3 levels of piRNA genes in piRNA clusters (1kb, centered on the piRNA TSS). The signal is represented as Z-scores. B) Metagene profile of H3K9me3 Z-score values across piRNA genes (1kb, centered on the piRNA TSS) located within H3K9me3 peaks. C) Metagene profile of H3K36me3 Z-score values across piRNA genes (1kb, centered on the piRNA TSS). (PDF) [file pgen.1011345.s001.pdf]

Supplemental Figure 2.

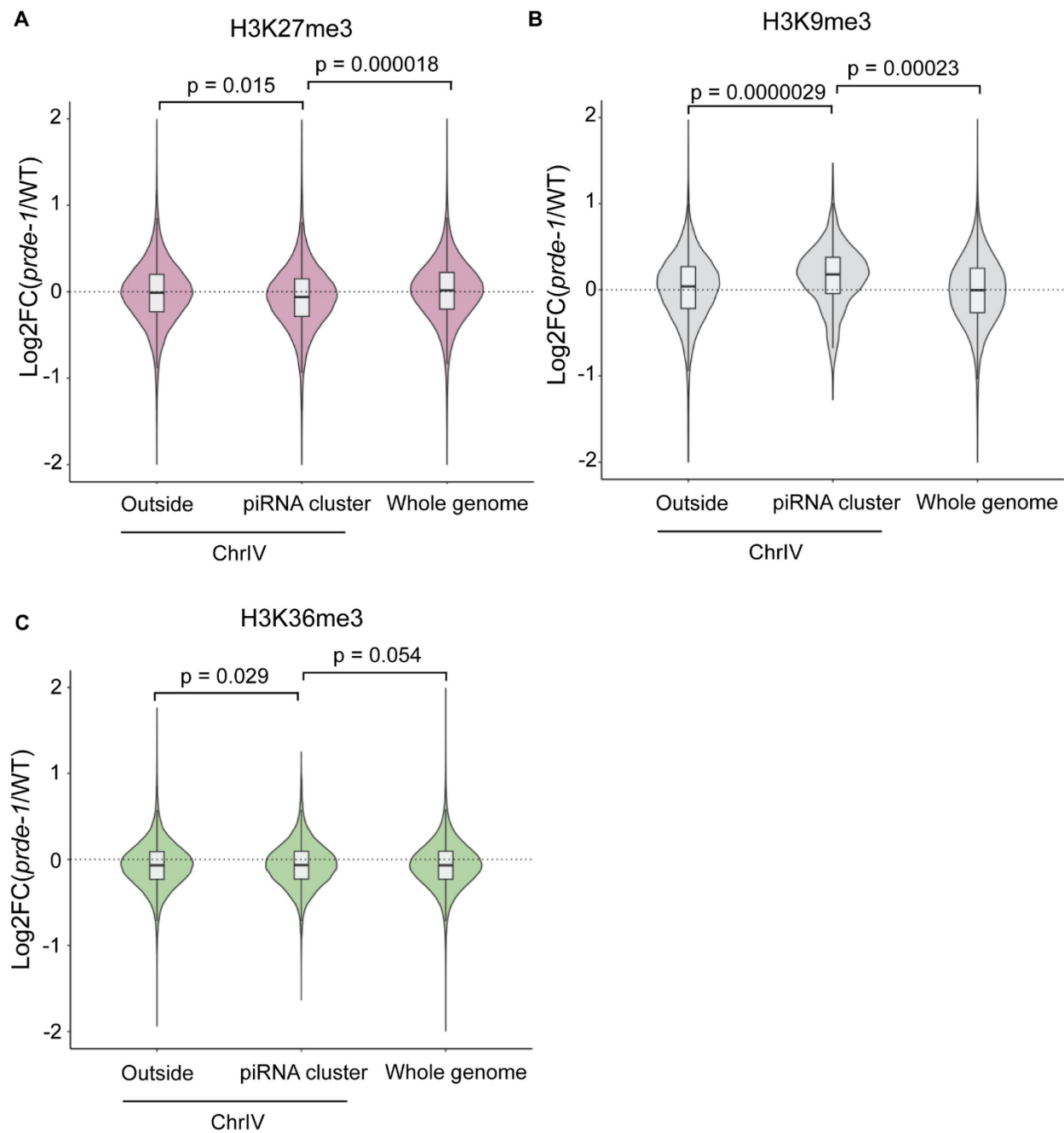

Supplement: S2 Fig — Histone modification violin plots for: A) H3K27me3 B) H3K9me3 C) H3K36me3. Violin plots showing log2fold change of histone modification signal at their respective peaks in prde-1 mutants relative to wild type. “Outside” refers to genomic regions on ChrIV that exclude piRNA gene cluster regions. “Whole genome” refers to genomic locations from all five autosomes and the X chromosome. (PDF) [file pgen.1011345.s002.pdf]

Supplemental Figure 3.

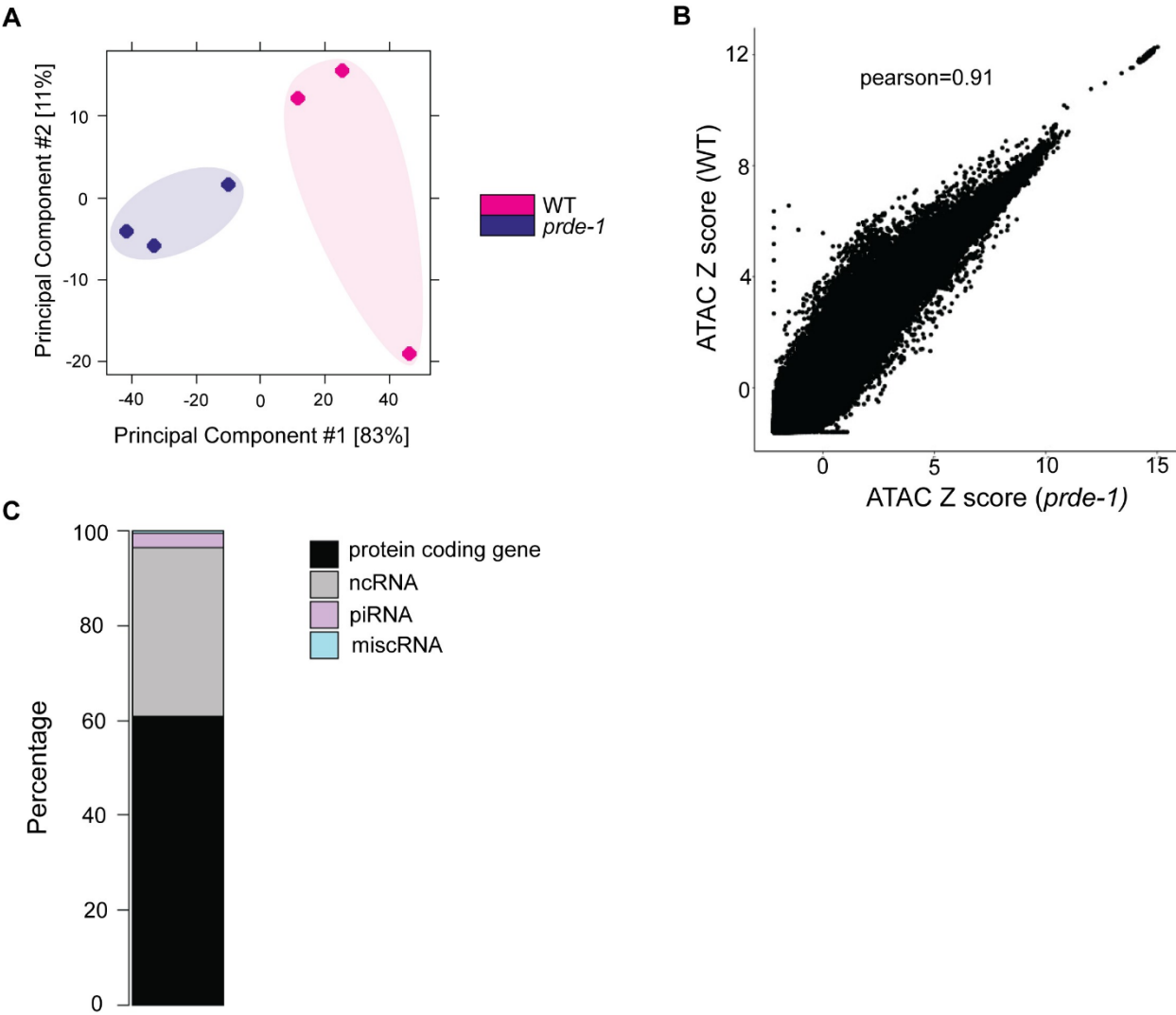

Supplement: S3 Fig — A) Principal component analysis of ATAC-seq replicates. Wild type—blue and prde-1 mutants—pink. B) Correlation scatterplot between wild type and prde-1 Z-score values, in 100bp bins. C) The y-axis of the stacked barplot represents percentage of associated genes from peaks significantly lost in the prde-1 mutant. (PDF) [file pgen.1011345.s003.pdf]

Supplemental Figure 4.

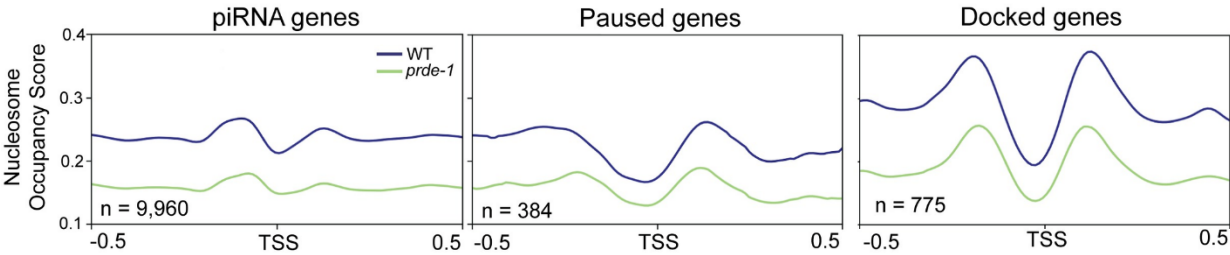

Supplement: S4 Fig — Metagene analysis of nucleosome occupancy scores (1kb, centered on the TSS of piRNA genes). Wild type values are in blue while prde-1 mutant values are in green for piRNA genes (left panel), RNA Pol II paused genes (middle panel), and RNA Pol II docked genes (right panel) [22]. (PDF) [file pgen.1011345.s004.pdf]

Supplemental Figure 5.

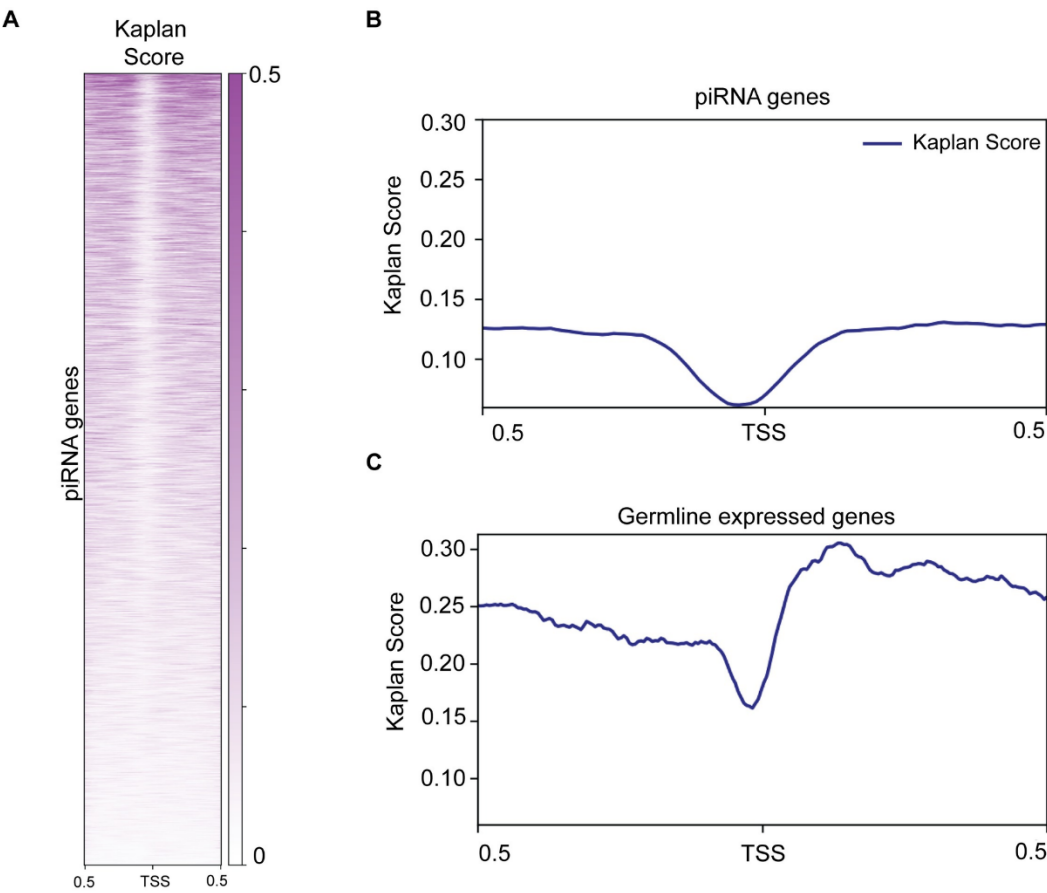

Supplement: S5 Fig — A) Heatmap representing Kaplan score centered at the TSS of piRNA genes. B) Metagene analysis of Kaplan score at piRNA genes. C) Kaplan score metagene analysis at germline-expressed genes centered at the TSS. (PDF) [file pgen.1011345.s005.pdf]

Supplemental Figure 6.

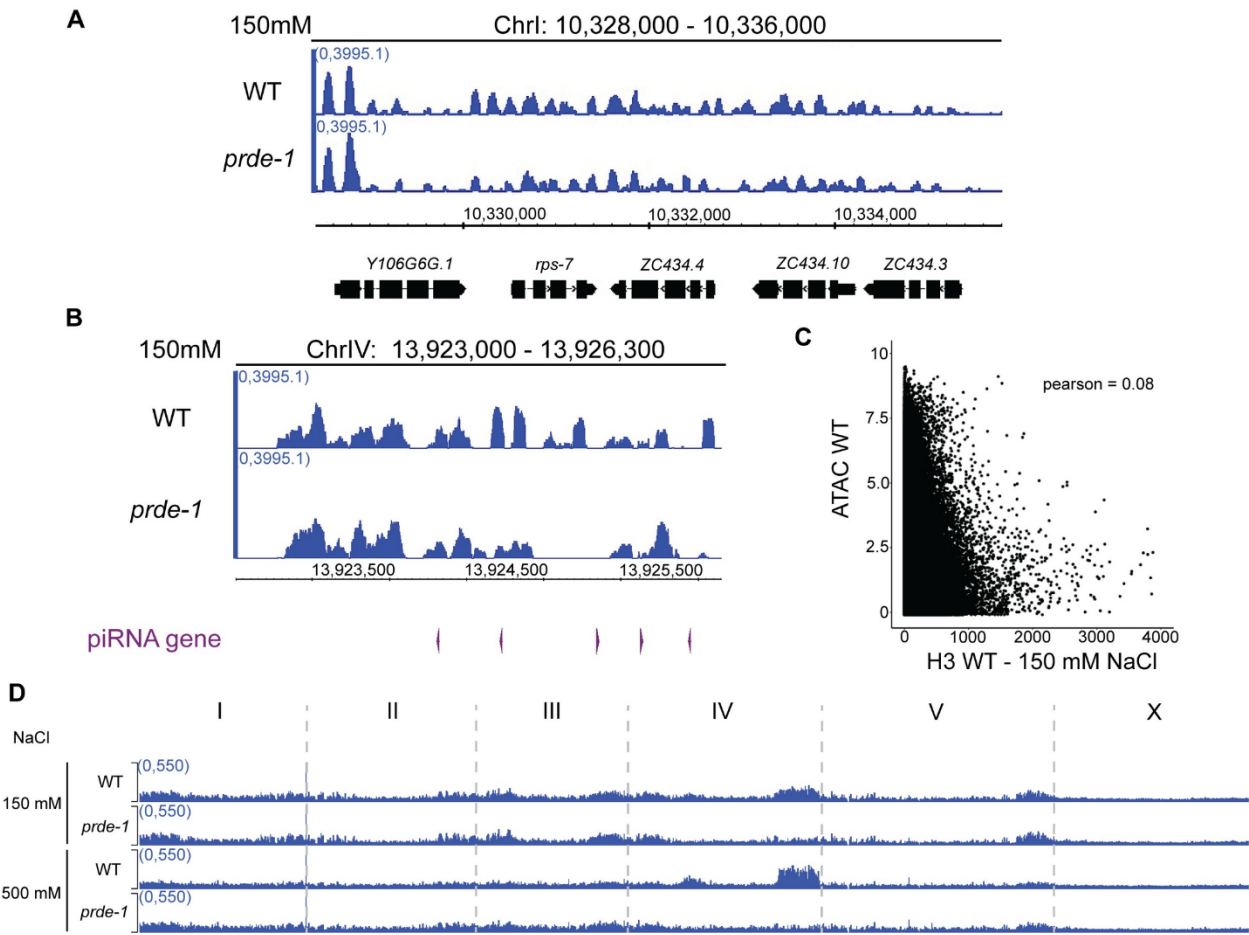

Supplement: S6 Fig — A-B) Genome browser shot within (A) and outside (B) the piRNA cluster under low salt conditions. C) Pearson correlation between ATAC-seq and low salt Native H3 ChIP-seq datasets, in 50bp bin. D) Genome browser shot of each individual chromosome of H3 signal in wild type and prde-1 mutants at low and high salt conditions. F) Quantification of upstream nucleosome occupancy score between WT and prde-1 mutant from DANPOS in low and high salt conditions. (PDF) [file pgen.1011345.s006.pdf]

Supplemental Figure 7.

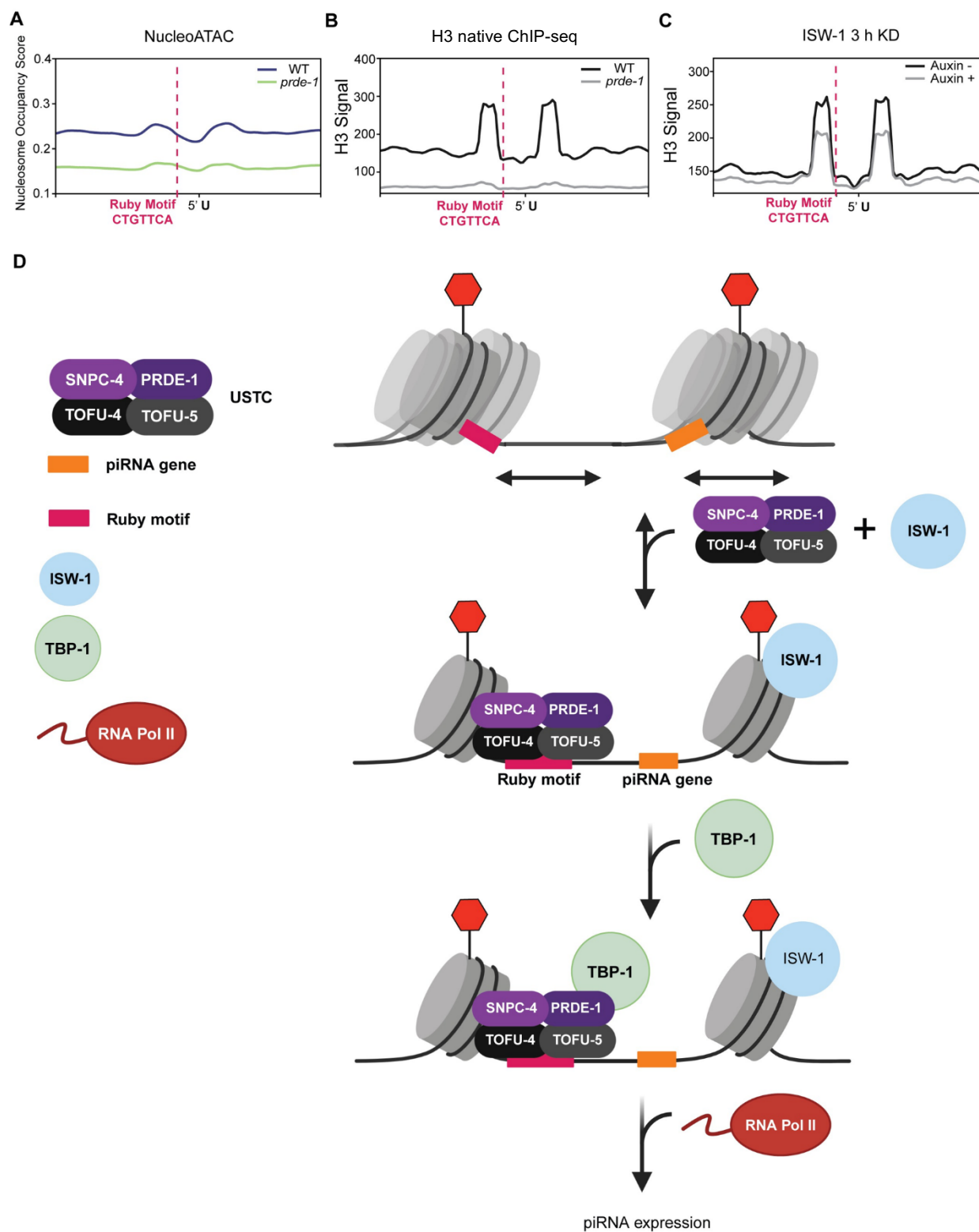

Supplement: S7 Fig — A) Nucleosome occupancy score at piRNA loci generated by NucleoATAC. B) H3 native ChIP-seq analysis at the piRNA locus in WT and prde-1 mutants. C) H3 native ChIP-seq after 3 h of ISW-1 KD vs ETOH control at piRNA locus. A-C) The magenta line represents 5’ end of RUBY motif and the 5’ U represents piRNA genes. D) Model of piRNA biogenesis. In the absence of ISW-1 and USTC, nucleosome positioning is highly variable, and regulatory sequences cannot be accessed. ISW-1 promotes nucleosome density and promotes USTC binding at the Ruby motif upstream of the piRNA TSS. Recruitment of TBP-1 by USTC then facilitates association of RNA polymerase II. (PDF) [file pgen.1011345.s007.pdf]
