# Supplementary material for: The Upstream Sequence Transcription Complex dictates nucleosome positioning and promoter accessibility at piRNA genes in the C. elegans germ line
Source: PLoS Genet. 2024 Jul 10;20(7):e1011345. doi: 10.1371/journal.pgen.1011345 (PMC11262695; doi:10.1371/journal.pgen.1011345)
Supplement: S8 Fig — A) Gblock sequence used for CRISPR-Cas9. B) crRNA sequence used for CRISPR-Cas9. C) All other primer sequences used in this study. (PDF) [file pgen.1011345.s008.pdf]

Supplemental Figure 8.

A. Gblock Repair Sequence.

Gblock sequence used as a template to generate *isw-1*::GFP::AID transgenic strain via the CRISPR-Cas9 System.

	Gblock sequence
GFP::AID::3xFlag	CTGTCGCTAAGGACCTGTCAAAGAGTAGTGGAACTCCAACGGCGAAAAAG GTAAAGGCTACTCCTAAAGGTGGCGGTGGATCGGGAGGAGGAGGTTCGA TGAGTAAAGGAGAAGAACTTTTCACTGGAGTTGTCCCAATTCTTGTTGAATT AGATGGTGATGTTAATGGGCACAAATTTTCTGTCAGTGGAGAGGGTGAGG GTGATGCAACATACGGAAAACTTACCCTTAAATTTATTTGCACTACTGGAAA ACTACCTGTTCCATGGGTAAGTTTAAACATATATATACTAACTAACCCTGAT TATTTAAATTTTCAGCCAACACTTGTCACTACTTTCTGTTATGGTGTTCAATG CTTCTCGAGATACCCAGATCATATGAAACAGCATGACTTTTTCAAGAGTGC CATGCCCGAAGGTTATGTACAGGAAAGAACTATATTTTTCAAAGATGACGG GAACTACAAGACACGTAAGTTTAAACAGTTCGGTACTAACTAACCATACATA TTTAAATTTTCAGGTGCTGAAGTCAAGTTTGAAGGTGATACCCTTGTTAATA GAATCGAGTTAAAAGGTATTGATTTTAAAGAAGATGGAAACATTCTTGGACA CAAATTGGAATACAACTATAACTCACACAATGTATACATCATGGCGGACAA ACAAAAGAATGGAATCAAAGTTGTAAGTTTAAACATGATTTTACTAACTAAC TAATCTGATTTAAATTTTCAGAACTTCAAAATTAGACACAACATTGAAGATG GAAGCGTTCAACTAGCAGACCATTATCAACAAAATACTCCAATTGGCGATG GCCCTGTCCTTTTACCAGACAACCATTACCTGTCCACACAATCTGCCCTTT CGAAAGATCCCAACGAAAAGAGAGACCACATGGTCCTTCTTGAGTTTGTAA CAGCTGCTGGGATTACACATGGCATGGATGAACTATACAAAGGATCCGGA GGTGGCGGGATGCCTAAAGATCCAGCCAAACCTCCGGCCAAGGCACAAG TTGTGGGATGGCCACCGGTGAGATCATACCGGAAGAACGTGATGGTTTCC TGCCAAAAATCAAGCGGTGGCCCGGAGGCGGCGGCGTTCGTGAAGGGAG GAGGATCCGACTACAAAGATCATGACGGTGATTATAAAGATCATGATATCG ATTACAAGGATGACGATGACAAGTAATTACATTCATATATTCTTTTTATCTCC TTCTCCATTCATTGCTTGATTAATGTCGATCTCTATCCTCCCTTTGATTACT GCTAAATCATCTCATCTCTCATCTTATCTCCTCATTTC

B. crRNA sequence

crRNA sequence targeting the endogenous *isw-1* locus using the CRISPR-Cas9 system.

crRNA	crRNA sequence
ISW-1 crRNA #1	GCTTTGACTTTCTTAGCAGTTGG

C. Primers used in this study.

Primer Name	Sequence
Forward primer for gblock amplification	CTGTCGCTAAGGACCTGTCAA
Reverse primer for gblock amplification	GAAATGAGGAGATAAGATGAGAGATGAGA
Forward primer for gblock without homology arm	CCAACGGCGAAAAAGGTAAAG
Reverse primer for gblock without homology arm	CTTGTCATCGTCATCCTTGTAATC
Forward genotype primer targeting insertion	ATGCCTAAAGATCCAGCCAAA
Reverse genotype primer targeting endogenous sequence	AAATAGCATAGGTAGGTGTGC
