# Supplementary material for: The Upstream Sequence Transcription Complex dictates nucleosome positioning and promoter accessibility at piRNA genes in the C. elegans germ line
Source: PLoS Genet. 2024 Jul 10;20(7):e1011345. doi: 10.1371/journal.pgen.1011345 (PMC11262695; doi:10.1371/journal.pgen.1011345)
Supplement: S1 Table — (DOCX) [file pgen.1011345.s009.docx]

**S1 Table. Strains used in this study**

| **Strain** | **Genotype** |
| --- | --- |
| JDW223 | wrdSi51 [mex-5p::TIR1::F2A::mTagBFP2::AID*::NLS::tbb-2 3'UTR] (II:0.77) |
| OP746 | unc-119(tm4063) III; wgIs746 [tbp-1::TY1::EGFP::3xFLAG + unc-119(+)] |
| SX2499 | prde-1(mj207) V |
| SX2650 | mjSi74 [mex-5p::wormCherry::prde-1::par-5] I |
| VC2010 | Wild type N2 |
| YL689 | mjSi74 [mex-5p::wormCherry::prde-1::par-5] I. unc-119(tm4063) III; wgIs746 [tbp-1::TY1::EGFP::3xFLAG + unc-119(+)] |
| YL691 | prde-1(mj207) V; wgIs746. |
| YL700 | ISW-1::eGFP::AID III |
| YL704 | mjSi74 [mex-5p::wormCherry::prde-1::par-5] I; wrdSi51 [mex-5p::TIR1::F2A::mTagBFP2::AID*::NLS::tbb-2 3'UTR] (II:0.77); ISW-1::eGFP::AID III |
